# The sickle cell trait affects contact dynamics and endothelial cell activation in *Plasmodium falciparum*-infected erythrocytes

**DOI:** 10.1038/s42003-018-0223-3

**Published:** 2018-11-30

**Authors:** Christine Lansche, Anil K. Dasanna, Katharina Quadt, Benjamin Fröhlich, Dimitris Missirlis, Marilou Tétard, Benoit Gamain, Bernd Buchholz, Cecilia P. Sanchez, Motomu Tanaka, Ulrich S. Schwarz, Michael Lanzer

**Affiliations:** 1 Department of Infectious Diseases, Parasitology, Universitätsklinikum Heidelberg, Im Neuenheimer Feld 324, 69120 Heidelberg, Germany; 2 Institute for Theoretical Physics and BioQuant-Center for Quantitative Biology, Heidelberg University, 69120 Heidelberg, Germany; 3 Physical Chemistry of Biosystems, University of Heidelberg, 69120 Heidelberg, Germany; 4 Department of Cellular Biophysics, Max Planck Institute for Medical Research, 69120 Heidelberg, Germany; 5 Université Sorbonne Paris Cité, Université Paris Diderot, Inserm, INTS, Unité Biologie Intégrée du Globule Rouge, UMR S1134, Severe Malaria Pathogenesis Group, Laboratoire d’Excellence GR-Ex, Paris, 75739 France; 6 Department of Hematology and Oncology, University Children’s Hospital, Medical Faculty Mannheim, 68167 Mannheim, Germany

**Keywords:** Computational models, Parasite host response, Malaria

## Abstract

Sickle cell trait, a common hereditary blood disorder, protects carriers from severe disease in infections with the human malaria parasite *Plasmodium falciparum*. Protection is associated with a reduced capacity of parasitized erythrocytes to cytoadhere to the microvascular endothelium and cause vaso-occlusive events. However, the underpinning cellular and biomechanical processes are only partly understood and the impact on endothelial cell activation is unclear. Here, we show, by combining quantitative flow chamber experiments with multiscale computer simulations of deformable cells in hydrodynamic flow, that parasitized erythrocytes containing the sickle cell haemoglobin displayed altered adhesion dynamics, resulting in restricted contact footprints on the endothelium. Main determinants were cell shape, knob density and membrane bending. As a consequence, the extent of endothelial cell activation was decreased. Our findings provide a quantitative understanding of how the sickle cell trait affects the dynamic cytoadhesion behavior of parasitized erythrocytes and, in turn, endothelial cell activation.

## Abstract (web summary)

Christine Lansche, Anil Dasanna et al. investigate the dynamic cytoadhesion behavior of erythrocytes infected with malaria parasite using quantitative flow chamber experiments and computer simulations. They find that parasitized erythrocytes have altered adhesion dynamics, mainly due to differences in cell shape, knob density and membrane bending.

## Introduction

Malaria has exerted a selective pressure on the human population for more than 200,000 years. Between 150,000 and 4000 years ago, genetic polymorphisms emerged in the human genome that bestow a survival benefit in infections with *Plasmodium falciparum*, the protozoan parasite causing the most virulent form of malaria in humans^1^. A prominent example of such a benefit trait is the sickle cell haemoglobin (HbS)^2^, which deviates from wild type haemoglobin (HbA) by a single amino acid substitution of valine for glutamic acid at position 6 in the ß-globin subunit of the haemoglobin hetero-tetramer. The mechanism by which the HbS and related structural haemoglobinopathies protect carriers from severe malaria is only partly understood. Recent developments point towards a close linkage with the mechanism by which *P. falciparum* causes severe disease^2^.

The virulence of *P. falciparum* is associated with the intraerythrocytic life cycle of the parasite and the altered haemodynamic properties of infected red blood cells. Whereas uninfected erythrocytes pass through the vascular system with the flow, parasitized erythrocytes develop cytoadhesive properties and sequester in the microvasculature to avoid passage through, and destruction in, the spleen^3^. Cytoadherence of parasitized erythrocytes can lead to vaso-occlusive events and impaired tissue perfusion^3^. These life-threatening complications are thought to be mitigated in carriers of the sickle cell trait as the corresponding parasitized erythrocytes display a reduced capacity to cytoadhere to microvascular endothelial cells^4^, although other mechanisms of protection are also being discussed, including modulation of the host’s immune response^5^, reduced intracellular multiplication under low oxygen tension^6^, and interference of parasite gene expression by host cell microRNA species^7^. Impaired cytoadhesion is associated with reduced amounts of surface-presented, parasite-encoded immuno-variant adhesins, collectively termed PfEMP1. The adhesins presented are displayed in abnormally enlarged and widely-dispersed membrane protrusions, termed knobs^4^.

Knobs are critical for dynamic and firm cytoadherence in flow^8^. Knobs concentrate the adhesin molecules, elevate them above the surface, and anchor them to the membrane skeleton for mechanical support under shear stress^9,10^. Knobs further stiffen the membrane by coupling it to the host cell’s spectrin/actin network and by causing strain hardening^11^. In the case of parasitized HbAS erythrocytes, there is evidence of an impaired interaction of the knobs with the host cell’s membrane skeleton and with a parasite-induced actin network required for vesicular trafficking of adhesins to the host cell surface^12,13^.

Dasanna et al. have recently simulated the effect of the knob distribution on cytoadhesion dynamics, using numerical simulations originally formulated to describe the rolling behavior of leukocytes^14^. They found that rolling of parasitized erythrocytes is favored by a homogeneous and fine-tuned knob distribution. Clustering knobs or varying the knob density to the extremes is detrimental for rolling and cause the cell to slip or arrest^14^. The drastic changes in knob architecture and density displayed by parasitized HbAS erythrocytes should, therefore, have a major impact on their mechanical and cytoadhesive properties. This, in turn, should affect not only firm cytoadherence^4,13^, but also dynamic interactions with the microvascular endothelium. Such effects might be further compounded by the intrinsic decreased cell deformability of erythrocytes carrying haemoglobin S^13,15,16^. Experimental data on the effect that altered cell mechanics has on adhesion dynamics of parasitized HbAS erythrocytes are scarce and a comprehensive and comparative quantitative description of the underpinning processes are not yet available. It is further unclear to what extent parasitized HbAS erythrocytes activate microvascular endothelial cells and how this process depends on the red blood cell-specific cellular and mechanical properties. Endothelial cell activation is thought to potentiate sequestration of parasitized erythrocytes and, thus, vascular obstruction through the upregulation and clustering of cytoadhesion receptors^17–20^.

Motivated by these considerations, we have compared the adhesion dynamics of parasitized wild type (HbAA) and HbAS erythrocytes in flow chamber experiments. We noted major differences, which we subsequently investigated in quantitative detail, using computer simulations based on a mathematical multiscale model for adhesive and deformable cells in hydrodynamic flow. This novel approach allowed us to identify cell shape, knob density, and membrane bending modulus as the main determinants for the different dynamic cytoadhesion behavior. Due to their differential biomechanical and cellular properties, parasitized HbAS erythrocytes made less contact per time unit and area with microvascular endothelial cells than did age-matched parasitized HbAA erythrocytes under comparable flow conditions. As a consequence, the extent to which parasitized HbAS erythrocytes activated microvascular endothelial cells was reduced. Our study provides novel, quantitative insights into the mechanism by which HbS protects carriers from severe malaria, by associating the altered cytoadhesion behavior and reduced endothelial cell activation with changes in biomechanical and cellular properties of parasitized HbAS erythrocytes.

## Results

### Distinct adhesion dynamics of parasitized HbAS erythrocytes

**Fig. 1:**
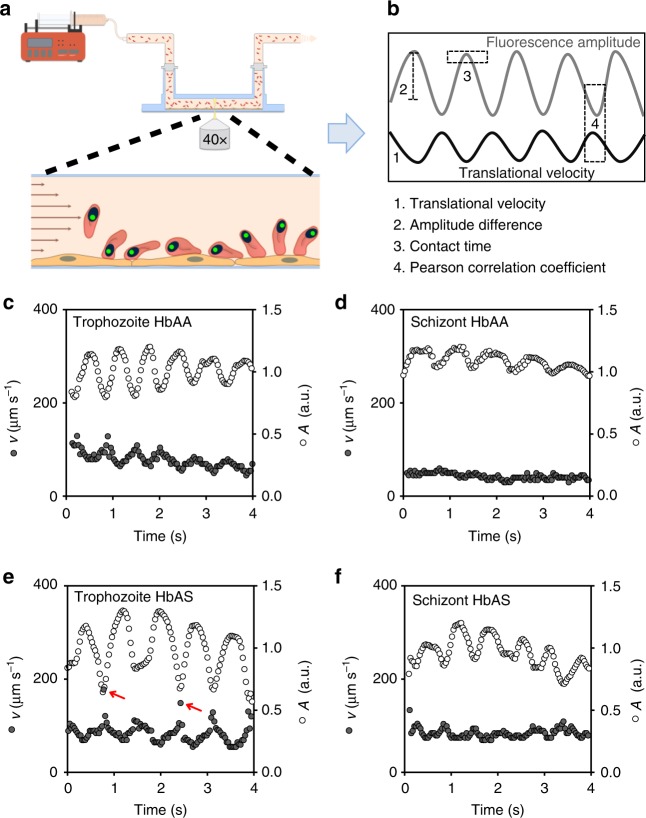
Stage-specific and erythrocyte variant-specific adhesion dynamics. **a** Schematic drawing of the experimental set up. *P. falciparum*-infected erythrocytes labeled with SYBR Green were superfused over a confluent human dermal microvascular endothelial cell monolayer at controlled hydrodynamic conditions. The motion behavior of infected erythrocytes was recorded by fluorescence microscopy, with the focal plane positioned on top of the endothelial cell monolayer. **b** Motion behavior of a single infected erythrocyte as defined by the fluorescence amplitude and the translational velocity trajectories. From the trajectories, the mean translational velocity, the amplitude difference, the contact time, and the Pearson correlation coefficient between the amplitude and the velocity trajectories were obtained. **c**–**f** Representative trajectories of the translational velocity, *v*, and fluorescence amplitude, *A*, of infected HbAA and HbAS erythrocytes at the trophozoite and schizont stage. The red arrows indicate velocity peaks, suggestive of a transient detachment of the parasitized HbAS erythrocyte from the substratum. The corresponding movies to the selected trajectories can be found in the supplementary information (Supplementary Movies 1-4). Wall shear stress, 0.03 Pa. a.u., arbitrary unit

We examined the adhesion dynamics of *P. falciparum*-infected erythrocytes on human dermal microvascular endothelial cells (HDMECs) in flow (Fig. 1a). HDMECs were chosen as a model substratum because they present CD36 and ICAM-1 (at high and low levels, respectively)^21^, and facilitate both dynamic and firm adhesive interactions with parasitized erythrocytes^22,23^. Prior to the flow chamber experiments, the *P. falciparum* strain FCR3 used throughout this study was repeatedly panned over HDMECs to enrich for a specifically cytoadhering population, henceforth termed FCR3^HDMEC^. Cytoadhesion to CD36 and ICAM-1 was confirmed using specific antibodies against these receptors, which blocked binding to HDMECs under flow conditions by 90% and 20%, respectively, when applied separately and by 99% when applied in combination (Supplementary Fig. 1). The selected parasite population predominantly expressed three PfEMP1-encoding *var* genes, namely IT4var13, IT4var25, and IT4var66 (Supplementary Fig. 2). The expressed *var* genes belong to the UpsB and UpsC subgroups and are predicted to confer binding to ICAM1 and/or CD36^24^.

To obtain quantitative data on cell motion, we labeled infected erythrocytes with the fluorescent DNA stain SYBR Green and followed the behavior of single cells, using automated imaging and tracking tools (Fig. 1). SYBR Green labeling did not affect the cytoadhesion efficiency of parasitized erythrocytes (Supplementary Fig. 3). For each cell, two parameters were obtained: the translational velocity and the fluorescence amplitude (Fig. 1b). Given that the parasite’s nucleus resides off-center in the host cell, the amplitude is a measure of the vertical movement of the parasite, as the cell moves over the endothelial cell monolayer, thereby moving in and out of the focal plane positioned on top of the endothelial cell monolayer (Fig. 1a, b).

We initially investigated highly synchronized parasitized erythrocytes at the trophozoite (26 ± 6 h post-invasion) and the schizont stage (36 ± 6 h post-invasion) in separate flow chamber experiments, with wall shear stresses ranging from 0.03 to 0.2 Pa, which represents physiological conditions found in venular microcapillaries^25^. For each hydrodynamic condition, we measured at least 27 cells from three independent biological replicates (Supplementary Fig. 4a and b).

Interestingly, the dynamic adhesion behavior of trophozoites and schizonts drastically differed. While trophozoites consistently displayed a flipping motion characterized by oscillating and anticorrelative velocity and amplitude trajectories, schizonts predominantly rolled over the endothelial monolayer, as indicated by an almost constant velocity trajectory and a lack of correlation between the velocity and the amplitude trajectory (Fig. 1c, d; Supplementary Fig. 5a and b and Supplementary Movies 1–12).

To systematically and quantitatively characterize the differences in adhesion dynamics, we parameterized the velocity and the amplitude trajectories and obtained the mean translational velocity, the mean amplitude difference, and the mean contact time with the substratum. In addition, we correlated the velocity trajectory with the amplitude trajectory for each cell, using a Pearson correlation.

**Fig. 2:**
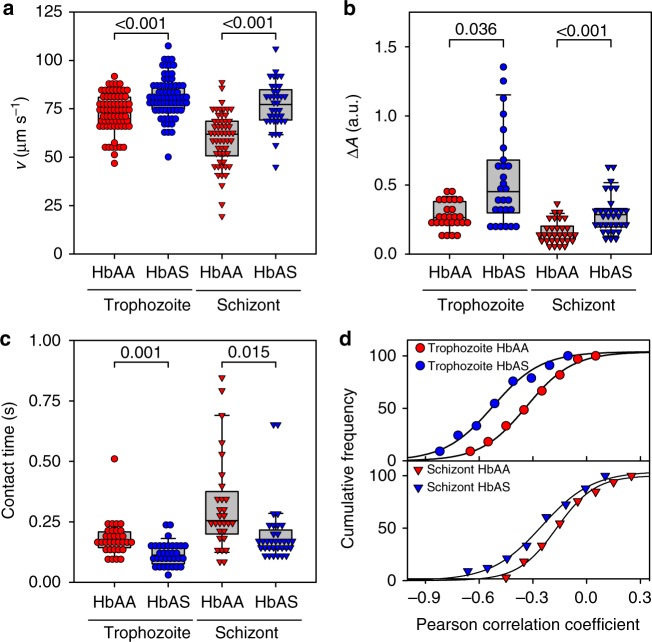
Quantitative analysis of the adhesion dynamics. The individual velocity and amplitude trajectories were parameterized and the following parameters were obtained: **a** the mean translational velocity, *v*; **b** fluorescence intensity amplitude difference, Δ*A*, (a.u., arbitrary units); **c** contact time; and **d** Pearson correlation coefficient between fluorescence amplitude and velocity profile. Note that the data for parasitized HbAA and HbAS erythrocytes are statistically different, according to an *F*-test (trophozoites: *F* = 58, DF = 21, *p* < 0.001; schizonts: *F* = 29, DF = 21, *p* < 0.001). Each data point corresponds to a single cell measurement, with at least 27 cells being measured per condition. A box plot analysis is overlaid over the individual data points, with the median, 25% and 75% quartile ranges and the standard error of the mean being shown. Statistical significance was assessed, using Holm–Sidak one-way ANOVA (velocity) or Dunn’s ANOVA on ranks (amplitude and contact time). Wall shear stress, 0.03 Pa

Although the overall number of cells that displayed dynamic adhesion behavior was comparable between parasitized HbAA and HbAS erythrocytes (unlike the number of firmly adhering cells that were reduced for HbAS erythrocytes^4,13^ (Supplementary Fig. 4)), there were clear differences in the dynamic adhesion behavior depending on the erythrocyte variant. At the trophozoite stage, parasitized HbAS erythrocytes frequently revealed sharp velocity peaks in regular intervals not found to the same extent in age and wall shear stress matched parasitized HbAA erythrocytes (Fig. 1c, e; Supplementary Fig. 5a and c and Supplementary Movies 1-12). The sharp peaks in translational velocity indicate a rapid flipping motion and/or a loss of contact with the substratum as the cell flipped over. In addition to the increased mean velocity (Fig. 2a and Supplementary Fig. 6a; *p* < 0.001 for both schizonts and trophozoites, according to Holm–Sidak one-way ANOVA), the mean amplitude difference was larger (Fig. 2b and Supplementary Fig. 6b; *p* = 0.036 and *p* < 0.001 for trophozoites and schizonts, respectively, according to Dunn’s ANOVA on ranks), whereas the contact time with the substratum was reduced (Fig. 2c and Supplementary Fig. 6c; *p* = 0.001 and *p* = 0.015 for trophozoites and schizonts, respectively, according to Dunn’s ANOVA on ranks), compared with parasitized HbAA erythrocytes. Similar differences in motion behavior were observed for the schizont stage (Fig. 1d, f). Here again parasitized HbAS erythrocytes displayed a higher mean velocity over the substratum (Fig. 2a and Supplementary Fig. 6a), indicating a faster rolling motion. However, the rolling was less smooth relative to age and wall shear stress matched parasitized HbAA erythrocytes (Fig. 1d, f), as indicated by the larger amplitude difference and the more pronounced anticorrelation between velocity and amplitude trajectories (Fig. 2b, d and Supplementary Fig. 6b and d). Overall, there was a more pronounced anticorrelation between the velocity and amplitude trajectories in parasitized HbAS erythrocytes at both the trophozoite and schizont stage than there was in parasitized HbAA erythrocytes, according to an *F*-test (trophozoites: *F* = 58, DF = 21, *p* < 0.001; schizonts: *F* = 29, DF = 21, *p* < 0.001) (Fig. 2d and Supplementary Fig. 6d).

The distinct motion behavior between infected HbAA and HbAS erythrocytes could not be explained by differences in parasite growth or the number of parasites per infected red blood cell. The FCR3^HDMEC^ strain grew in both red blood cell types with comparable intraerythrocytic development rates, multiplication rates, and multiple infectivity (Supplementary Fig. 7). Therefore, we set out to systematically identify the main factors causing the different adhesion dynamics.

### Effect of cell shape on adhesion dynamics

**Fig. 3:**
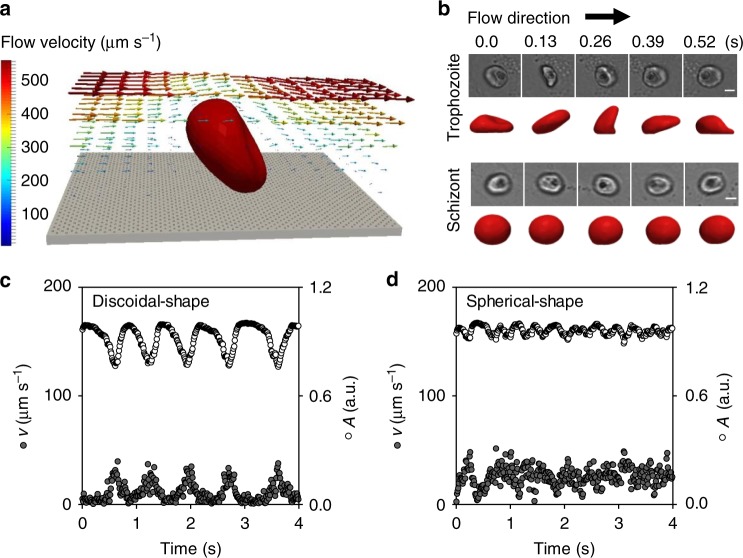
Effect of cell shape on dynamic cytoadhesive behavior. **a** Schematic representation of the mesoscopic computer simulation, based on a deformable red blood cell model and multiparticle collision hydrodynamics, used to assess the adhesion dynamics of parasitized erythrocytes. Fluid flow lines derived from the simulation are shown around a flipping cell. Flow velocities are indicated by a color code. **b** Representative time series of a flipping trophozoite and a rolling schizont are shown. The upper panels show the motion behavior as recorded by phase contrast microscopy (see also Supplementary Movies 5 and 6). The lower panels show the corresponding mesoscopic simulations (see also Supplementary Movies 7 and 8). The time points at which the snapshots were recorded are indicated. Wall shear stress, 0.03 Pa; scale bar, 5 µm. **c**, **d** Simulation of the fluorescence amplitude, *A*, and velocity trajectory, *v*, of **c** a flipping cell with an asymmetric discoidal shape and **d** a rolling cell with an almost spherical shape. a.u., arbitrary units

For a quantitative analysis of the relevant factors, we turned to computer simulations, using a deformable red blood cell model and multiparticle collision hydrodynamics (Fig. 3a)^26,27^. We initially focused on the effect of the cell shape since this parameter drastically changes from biconcave discoidal to almost spherical as the parasite matures during the 48 h intraerythrocytic developmental cycle^28^. Quantitative data on cell shape and volume were taken from Waldecker et al. who measured geometric cell parameters in intervals of 4 h throughout the 48 h replicative cycle for the FCR3 strain^28^. For the simulations, we considered two cell shapes: a discoidal form with a volume of 79 µm^3^ and a reduced volume of 0.79 as is displayed by trophozoites and an almost spherical form with a volume of 114 µm^3^ and a reduced volume of 0.99 as is found for late schizonts. The membrane shear and bending moduli were set at values of 20 µN m^−1^ and 85 *k*_B_*T*, respectively, as determined for parasitized HbAA erythrocytes at the trophozoite stage^29^. The simulations were performed using a wall shear stress of 0.1 Pa. The kinetic rates were tuned to *κ*_on_ ≃ 6 Hz and *κ*_off_ ≃ 18 Hz for the PfEMP1/receptor interactions. The knob density was set at 5 knobs µm^−2^, as determined by atomic force microscopy (Supplementary Fig. 8)^30^. We further approximated the intracellular parasite as a rigid sphere of 3 µm^3^. As seen in Fig. 3b, changing the cell shape from a biconcave discoid to a sphere drastically affected the motion behavior and altered it from flipping to rolling. The simulations further fully reproduced the anti-correlation between the translational velocity and the fluorescence amplitude in trophozoites and the reduction of this correlation in schizonts (Fig. 3c, d). Thus, the differential dynamic adhesion behavior of trophozoites and schizonts is a direct result of their different cell shapes^14,26^. At very high shear rates, the initial cell shape no longer plays a dominant role since the cells increasingly deform into comparable shapes. The decrease in the fluorescence amplitude differences from the trophozoite to the schizont stage seen in flow chamber experiments (Fig. 1c, d; and Supplementary Fig. 5a and b), reflects the gradual occupation of the entire host cell volume by the growing parasite^28^.

### Effect of cell stiffness and knob density on adhesion

**Fig. 4:**
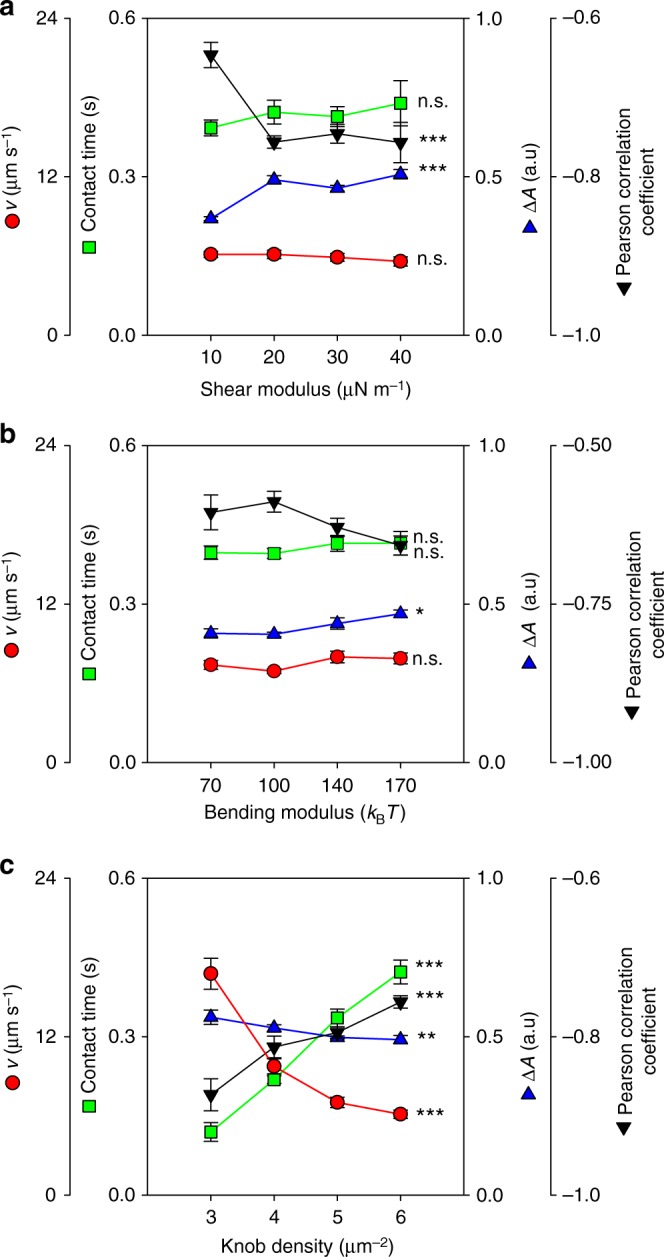
Effect of membrane stiffness and knob density on dynamic cytoadhesive behavior. Simulations were performed to assess the effect of **a** the shear modulus, **b** bending modulus, and **c** knob density on adhesion dynamics of parasitized erythrocytes. As readouts the following parameters were obtained: translational velocity, *v*, contact time, fluorescence amplitude difference, Δ*A*, (a.u., arbitrary units), and the Pearson correlation coefficient between fluorescence amplitude and velocity trajectories. The simulations were performed for a cell with a discoidal shape (absolute volume of 79 µm^3^ and reduced volume of 0.79) and a wall shear stress of 0.1 Pa. The shear and bending moduli considered cover the range of values experimentally determined for infected and uninfected erythrocytes^29^. The range of knob densities cover the values experimentally determined for HbAA and HbAS erythrocytes infected with the FCR3^HDMEC^ strain (Supplementary Fig. 8). Statistical significance was assessed using Holm–Sidak one-way ANOVA. n.s., not significant. *p < 0.05; **p < 0.01; ***p < 0.001

We next considered the effect of the cell stiffness on adhesion dynamics and varied the shear modulus and the bending modulus in separate computer simulations, while keeping the other variables constant at the values described above and by assuming a cell shape as displayed by trophozoites. The selected shear moduli ranged from 10 to 40 µN m^−1^ and the selected bending moduli from 70 to 170 *k*_B_*T*, covering the range of values experimentally determined for trophozoites and schizonts in optical tweezer experiments^29^. Figure 4a, b shows that the fluorescence amplitude increased with rising shear or bending modulus, whereas the Pearson correlation between the velocity and amplitude decreased. The translational velocity and the contact time were largely unaffected by changes in the shear and bending modulus. These findings suggest that a stiffer plasma membrane causes the parasitized erythrocyte to flip more regularly, hence, the more negative Pearson correlation, than a cell with a softer membrane, which is more prone to elastic deformation in flow.

We then simulated the effect of the knob density on the transient adhesion behavior of parasitized erythrocytes at the trophozoite stage. The bending and shear modulus were fixed at values of 85 *k*_B_*T* and 20 µN m^−1^. All other parameters were as described above. As shown in Fig. 4c, the translational velocity and the fluorescence amplitude strongly decreased with increasing knob density, whereas the contact time and the Pearson correlation coefficient increased. These findings are plausible since the number of knobs is directly proportional to the number of receptor/ligand interactions^14^.

### Modeling adhesion dynamics

We next turned to the differential adhesion dynamics displayed by infected HbAA and HbAS erythrocytes and fed into our simulation experimentally-determined parameters for these two cell types. Parasitized HbAS erythrocytes had fewer knobs than did age-matched infected HbAA erythrocytes (3 ± 1 and 5 ± 1 knobs µm^−2^ for trophozoites and 5 ± 1 and 8 ± 1 for schizonts), and the knobs present were mostly enlarged (Supplementary Fig. 8)^4^. The membrane bending modulus was determined for the trophozoite stage, using flicker spectroscopy, and yielded values of 84 ± 34 and 143 ± 29 *k*_B_*T* (*p* < 0.001 according to *t*-test) for parasitized HbAA and HbAS erythrocytes, respectively, consistent with parasitized HbAS erythrocytes having a stiffer host cell plasma membrane. The volumetric parameters were as following: 79 and 70 µm^3^ (reduced volume of 0.79 and 0.66) for infected HbAA and HbAS erythrocytes at the trophozoite stage and, for schizonts, 114 and 70 µm^3^ (reduced volume of 0.99 and 0.75), respectively^28^. The smaller volumetric values of parasitized HbAS erythrocytes arise from microvesiculation and delayed osmotic swelling^28,31^. All other parameters were as described above.

**Fig. 5:**
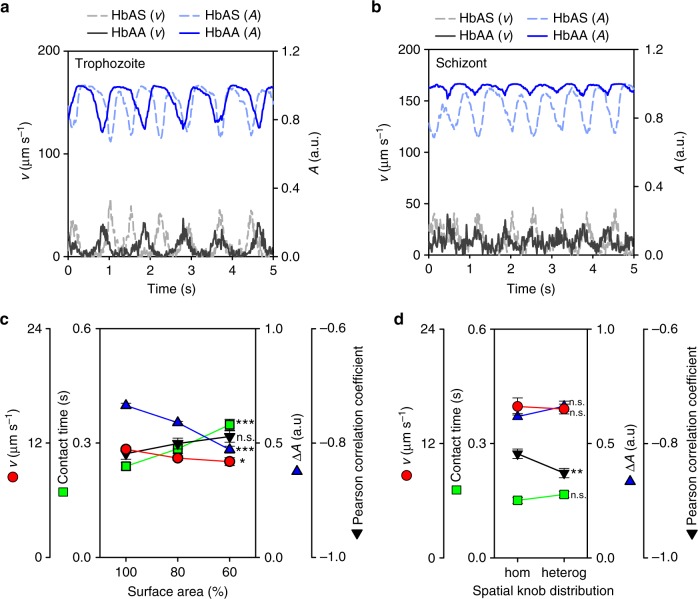
Simulated trajectories of parasitized HbAA and HbAS erythrocytes. Representative translational velocity, *v*, and fluorescence amplitude trajectories, *A*, are shown for parasitized HbAA and HbAS erythrocytes at **a** the trophozoite and **b** schizont stage. The following experimentally determined parameters were used for the simulations, with the parameters given in the following order: reduced volume, knob density, bending modulus, and shear modulus. HbAA trophozoites, 0.79, 5 knobs µm^−2^, 84 *k*_B_*T*, 20 µN m^−1^; HbAS trophozoites, 0.66, 3 knobs µm^−2^, 143 *k*_B_*T*, 40 µN m^−1^; HbAA schizonts, 0.99, 8 knobs µm^−2^, 84 *k*_B_*T*, 20 µN m^−1^; HbAS schizonts, 0.75, 5 knobs µm^−2^, 143 *k*_B_*T*, 40 µN m^−1^. Wall shear stress, 0.1 Pa. **c** Effect of cell size on the dynamic adhesion of infected erythrocytes. Three different cell sizes were selected: 100%, 80%, and 60% of surface area. The translational velocity, amplitude difference, contact time, and Pearson correlation coefficient between fluorescence amplitude and velocity trajectories are shown for a trophozoite. The knob density was kept constant in all the cases and a bending modulus and a shear modulus of 84 *k*_B_*T* and 20 µN m^−1^, respectively, were considered. Wall shear stress, 0.1 Pa. **d** Effect of the knob distribution on adhesion dynamics. A homogeneous knob distribution (hom) was simulated by keeping the distance between any two consecutive knobs almost the same, whereas the heterogeneous knob distribution (heterog) represents our standard approach as described in the methods section. All other parameters were as described above. Statistical significance was assessed using Holm–Sidak one-way ANOVA (**c**) or Student’s two-tailed *t*-test (**d**). n.s., not significant. *p < 0.05; **p < 0.01; ***p < 0.001

The simulations yielded velocity and amplitude trajectory profiles (Fig. 5a, b), which were in good agreement with the results of our flow chamber experiments. This included the sharp velocity peaks and the pronounced anticorrelation between velocity and amplitude trajectories in parasitized HbAS erythrocytes at the trophozoite stage and, at the schizont stage, the bumpy rolling phenotype (compare Fig. 1). Changes in cell size (realized as changes of surface area at constant reduced volume) did not affect much the adhesion dynamics of parasitized HbAS erythrocytes because shear and adhesion forces change in a similar manner with cell size (Fig. 5c). In our simulations, we distributed the knobs randomly over the surface, thus representing the natural heterogeneity. A more homogenous knob distribution did not have a large effect (Fig. 5d). We conclude that the reduced knob density in HbAS erythrocytes results in a larger velocity and a shorter contact time, whereas the stiffer membrane affects mainly the amplitude changes.

**Fig. 6:**
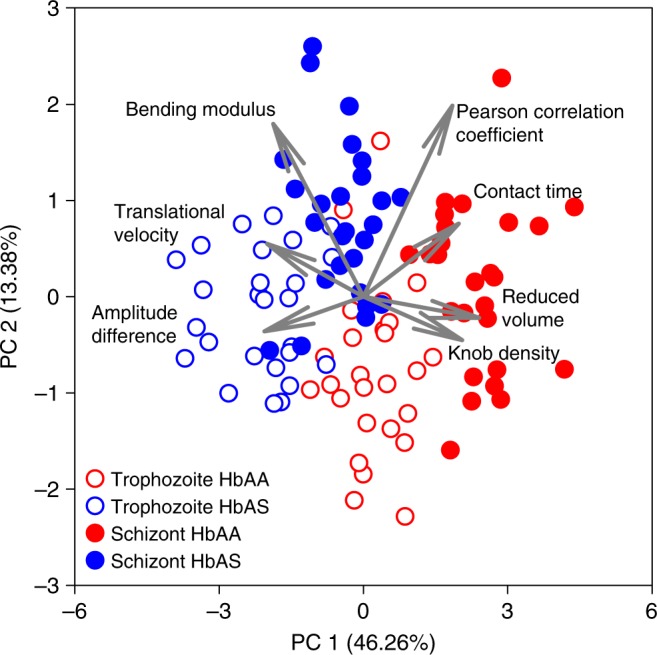
Principal component analysis of adhesion dynamics of parasitized HbAA and HbAS erythrocytes. The score plot of the first two principal components (PC1 and PC2) is shown and the percentage of the total variance explained by each principal component is given in parenthesis. The combined principal components PC1 and PC2 explain 59.64% of the variance in the data. The plot is overlaid with the eigenvectors (gray arrows) whose length and direction indicate how influential a variable is. Each dot represents the data set obtained from a single cell

To corroborate these conclusions we performed a principal component analysis, using the empirically-determined parameters (knob density, reduced volume, translational velocity, amplitude difference, Pearson correlation coefficient between translational and amplitude trajectory, contact time, and bending modulus) as variables (Fig. 6). It is evident from the score plot that the amplitude difference and the contact time are anti-correlated as are knob density and velocity (Fig. 6). Interestingly, the principal component analysis separated the cells according to stage and erythrocyte variant, with each condition occupying a different quadrant in the score plot (Fig. 6). The lower left quadrant is dominated by a large amplitude difference, a high translational velocity and a low knob density, and it is here where the HbAS erythrocytes at the trophozoite stage cluster. The upper left quadrant, which is governed by a high bending modulus contains the HbAS schizonts. In comparison, HbAA erythrocytes at the trophozoite and schizont stage group in the lower right and upper right quadrant, respectively. Their behavior is dominated by a low bending modulus (trophozoites) and, in the case of schizonts, by a high knob density, a high contact time, a reduced volume approaching a sphere, and a low translational velocity. Thus, the principal component analysis of the experimental data validated the predictions made by the simulations, and showed that parasitized HbAA and HbAS erythrocytes but also trophozoites and schizonts can be clearly separated from one another according to their adhesion dynamics.

### Erythrocyte variant specific footprints

**Fig. 7:**
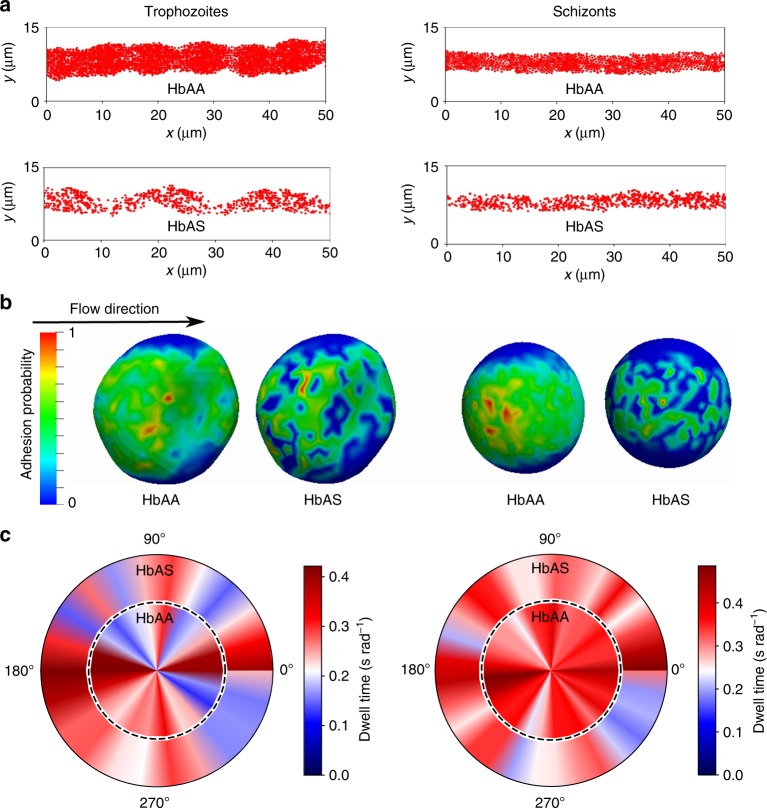
Contact interactions between parasitized erythrocytes and human dermal microvascular endothelial cells. **a** Representative examples of simulated contact footprints that parasitized HbAA and HbAS erythrocytes at the trophozoite and schizont stage leave on the substratum. For simulation parameters see Fig. 5 legend, except that, for comparative reasons, a spherical shape with a reduced volume of 0.99 was used for both parasitized HbAA and HbAS erythrocytes at the schizont stage. **b** Representative simulated adhesive patches are shown for parasitized HbAA and HbAS erythrocytes at the trophozoite and schizont stage. The color code indicates the relative number of productive cytoadhesive interactions between the parasitized erythrocyte and the substratum at a particular point in time. The cells are viewed from the side facing the substratum. **c** Experimentally determined adhesive contact maps of trophozoites and schizonts. The adhesive contact maps were obtained by projecting the amplitude profiles derived from the flow chamber experiments around the circumference of the cells, yielding an average dwell time per unit angle as an indicator of contact points between the cell and the substratum at a given spatial and temporal position

To explore putative physiological ramifications of the differential adhesion behavior, we next simulated the footprint that parasitized HbAA and HbAS erythrocytes might leave on a substrate. Interestingly, the footprints differed considerably between the different parasite stages and different red blood cell variants (Fig. 7). First, the footprints became narrower with less contact points as the parasite developed from trophozoite to schizont (Fig. 7a). These findings are explained by the change in cell shape from discoidal to spherical and a concomitant loss in membrane elasticity. Thus, in schizonts, contact with the substratum is made only in a small band along the circumference (Fig. 7b). This small band of contact shrinks in parasitized HbAS erythrocytes due to the stiffer cell membrane (Fig. 7a). Second, parasitized HbAS erythrocytes at the trophozoite stage display footprints that widened and narrowed in regular intervals (Fig. 7a) and, at higher wall shear stresses, even tear off and become patchy (Supplementary Fig. 9). In comparison, the footprint of parasitized HbAA erythrocytes is continuous at all stages and all hydrodynamic conditions investigated (Fig. 7a and Supplementary Fig. 9).

To further characterize the predicted footprints, we projected the amplitude profiles derived from the flow chamber experiments around the circumference of the cells and obtained an average dwell time per unit angle^32^. In the case of trophozoites, areas of tight contact alternated with areas of loose contact, with little distinction between the red blood cell variants (Fig. 7c). Such a pattern is consistent with a flipping motion and an oscillating footprint. A comparable pattern was found for parasitized HbAS erythrocytes at the schizont stage. Here again areas of tight contact alternated with areas of loose contact, which would suggest a bumpy rolling phenotype and a reduced number of contact points with the substratum, consistent with the flow chamber experiments and the simulated footprint. In comparison, parasitized HbAA erythrocytes were continuously in contact with the substratum (Fig. 7c), consistent with a smooth rolling motion and a continuous footprint.

### Differential endothelial cell activation

**Fig. 8:**
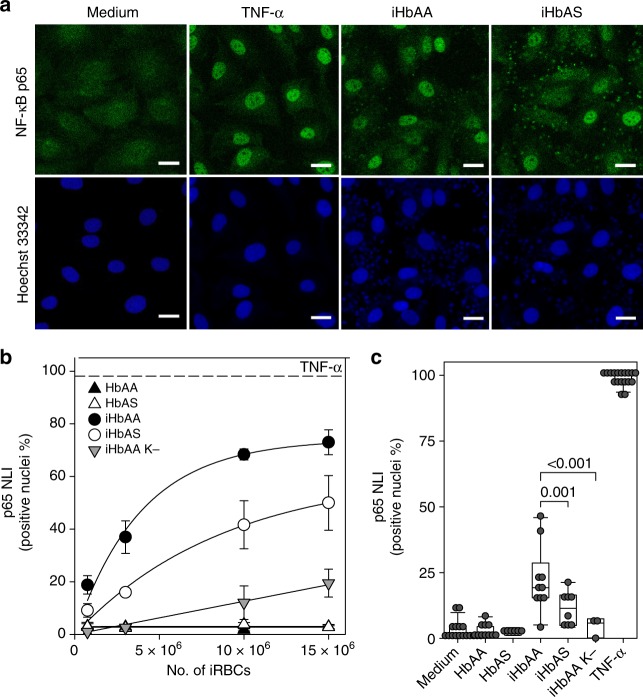
Reduced endothelial cell activation by infected HbAS erythrocytes. **a** Representative confocal microscopy images showing the subcellular localization of p65-NFĸB subunit in human dermal microvascular endothelial cells (HDMEC) upon TNF-α treatment (100 units ml^−1^ for 2 h) or upon co-culture with parasitized HbAA or HbAS erythrocytes (1 × 10^7^ infected red blood cells at the trophozoite stage for 2 h) under static conditions. Medium served as a negative control. The p65-NFĸB subunit was localized using a specific polyclonal rabbit antiserum (dilution 1:200) and an Alexa-488 conjugated goat anti-rabbit IgG antiserum (1:400) as secondary antibody. The nuclei were stained using Hoechst 33342. Scale bar, 20 µm. **b** Percentage of HDMECs positive for nuclear p65-NFĸB staining (nuclear labeling index, NLI) as a function of the parasite load under static conditions. Infected HbAA and HbAS erythrocytes (iHbAA and iHbAS) were analyzed. TNF-α served as a positive control and uninfected HbAA and HbAS red blood cells (HbAA and HbAS) were used as negative controls. As an additional control, the effect of an isogenic knobless FCR3 line (iHbAA K−) was investigated. The mean ± SEM of at least three independent biological replicates is shown, with at least 50 endothelial cells being analyzed per condition and per biological replicate. Note the data obtained for parasitized HbAA and HbAS as well as for the HbAA erythrocytes infected with the knobless parasite line are significantly different, as determined using *F*-tests (*F* = 28; DF = 4; *p* < 0.001 and *F* = 169; DF = 4; *p* < 0.001, respectively). **c** Endothelial cell activation under flow conditions. Infected and uninfected HbAA and HbAS erythrocytes (1 × 10^8^ cells) were superfused over a confluent monolayer of HDMECs at a wall shear stress of 0.03 Pa. A box plot analysis is overlaid over the individual data points, with the median, 25% and 75% quartile ranges and the standard error of the mean being shown. At least 50 endothelial cells were analyzed per condition and per biological replicate. Statistical significance was determined using one-way ANOVA

We finally investigated the effect of the differential cytoadhesion behavior on microvascular endothelial cell activation under static and flow conditions. As a common denominator of endothelial cell activation, we monitored the translocation of the NF-ĸB subunit p65 from the cytoplasm to the nucleus by immunofluorescence microscopy, using a polyclonal antibody against p65^17^. To this end, an HDMEC monolayer was co-cultured with increasing amounts of parasitized erythrocytes ranging from 7.5 × 10^5^ to 1.5 × 10^7^ cells (trophozoites) for 2 h under static conditions before the p65-associated nuclear fluorescence signal was determined. As a positive control, we stimulated the endothelial cells with TNF-α (100 units ml^−1^), which resulted in 100% of the cells being activated (Fig. 8a). Both parasitized HbAA and HbAS erythrocytes activated the endothelial cells in a concentration-dependent manner (Fig. 8a, b). However, the extent of activation depended on the erythrocyte variant, with the number of activated endothelial cells being significantly higher for parasitized HbAA erythrocytes, as compared with parasitized HbAS erythrocytes, at all conditions tested (*F* = 28, DF = 4, *p* < 0.001, according to an *F*-test). Uninfected HbAA and HbAS erythrocytes investigated in parallel assays did not stimulate the endothelial cells and an isogenic knobless FCR3 line had only a marginal effect on endothelial cell activation (Fig. 8b). Furthermore, the addition of purified human ICAM-1 and/or CD36 (50 µg ml^−1^) to the assay reduced endothelial activation by parasitized HbAA erythrocytes by approximately 70% (Supplementary Fig. 10a). In control experiments using trans-well plates, we confirmed that the activation of endothelial cells required cell-to-cell contact and did not occur when endothelial cells and infected erythrocytes were physically separated from one another by a permeable membrane^33^ (Supplementary Fig. 10b and c).

We repeated the experiments, but this time under flow conditions in which 1 × 10^8^ infected erythrocytes at the trophozoite stage were flushed through the flow chamber at a wall shear stress of 0.03 Pa. Here again, infected HbAA erythrocytes activated significantly more endothelial cells than did infected HbAS erythrocytes (*p* = 0.001, according to Holm–Sidak one-way ANOVA) (Fig. 8c). Uninfected red blood cells and the knobless FCR3 line served as negative controls and TNF-α as a positive control (Fig. 8c).

## Discussion

Sickle cell trait protects carriers from severe malaria via a mechanism that involves reduced cytoadherence of parasitized HbAS erythrocytes to microvascular endothelial cells, as has been shown mainly by measuring the number of firmly adhering cells^4,13^. Our study extends this conclusion by demonstrating that the sickle cell trait also affects the dynamic adhesive interactions with the endothelium. Parasitized HbAS erythrocytes flipped and rolled faster over the endothelial cells at the trophozoite and schizont stage, respectively, and the contact time and contact area were reduced, as compared with age-matched parasitized HbAA erythrocytes under comparable hydrodynamic conditions (Figs. 2 and 7). The main determinants of these differential adhesion dynamics were cell shape, knob density, and cell mechanics.

Erythrocytes containing the HbS suffer from an intrinsic oxidative stress, owing to an increased reactivity of haemoglobin S with oxygen and the subsequent formation of irreversibly oxidized haemichromes and reactive iron species^13,16,34^. These highly reactive oxidants interfere with numerous physiological processes of the red blood cell, including ion homeostasis, membrane stability, membrane skeletal organization, and viscoelasticity^16^. Infection with *P. falciparum* aggravates the oxidative stress^35^, with consequences for knob formation, solute pathways, cell geometry, membrane elasticity, and host actin remodeling^13,28^. For instance, parasitized HbAS erythrocytes have fewer but enlarged knobs^4,13^; their membrane is stiffened as indicated by a higher bending modulus; they can lose surface area^28^; and their cell volume does not approach a spherical shape at the end of schizogeny as is seen for parasitized HbAA erythrocytes, but remains biconcaved due to a delayed activation of the new permeation pathways^28^. A stiffening of the membrane is also observed in parasitized HbAA erythrocytes^11,29^, but not to the same extent.

The computational simulations allowed us to quantitatively assess the contribution of each of the different factors. The knob density inversely correlated with the translational velocity and directly with the contact time (Fig. 6). The flexural moduli affected mainly the vertical cell movement (Fig. 4). The latter is plausible given that a cell with a stiff membrane can resist the shear stress and does not bend until the point when it flips over, hence the larger fluorescence amplitude as compared with a cell with a soft membrane. Consistent with this conclusion, previous computer simulations on uninfected erythrocytes showed that a cell with a low elastic modulus crawls on a surface, whereas a cell with a higher elastic modulus switches to a flipping motion^26^. On the basis of these considerations, we explain the rolling phenotype of the schizonts by their almost spherical cell shape, a stiff membrane and a high knob density. In comparison, the flipping motion displayed by trophozoites is largely due to their biconcave shape. The differences in cell morphology and mechanics between schizonts and trophozoites further affected the translational velocity at which these cells moved over the substratum and the time period of interaction with the endothelium (Figs. 2 and 7). Trophozoites moved at high translational velocity as they cytoadhered for only short periods of time, while experiencing large displacements during flipping transitions (Figs. 1 and 2). In comparison, the rolling motion of schizonts was characterized by a much slower translational velocity, small cell displacements and longer contact times (Figs. 1 and 2). Although schizonts are unlikely to be recruited from flow, we included schizonts in the study to demonstrate the effect of the cell shape, cell rigidity, and knob density on adhesion dynamics.

Feeding the relevant parameters for the knob density, the cell shape and the bending modulus into our simulations phenotypically reproduced the differential adhesion dynamics displayed by parasitized HbAS erythrocytes. This included the pronounced flipping motion at the trophozoites stage and the rough rolling at the schizont stage, as indicated by higher mean velocities, larger fluorescence amplitude differences, shorter contact times, and more negative Pearson correlation coefficients between fluorescence amplitude and translation velocity trajectories (compare Figs. 1 and 5). Thus, the rough rolling phenotype of infected HbAS erythrocytes at the schizont stage resulted from the non-spherical cell shape, a low knob density, and a stiff membrane. Similarly, the cell shape, the high bending modulus, and the lower knob density can largely account for the pronounced flipping motion at the trophozoite stage (compare Figs. 1 and 5).

The simulations further corroborated experimental data, suggesting that parasitized HbAS erythrocytes leave a smaller footprint, as defined by the number of contact points, on endothelial cells than do infected HbAA erythrocytes (Fig. 7). While the simulations predicted differences in the translational velocity between infected HbAA and HbAS erythrocytes, they failed to forecast the absolute values, with the simulations consistently falling short. Possible reasons are the specifics and variability of the receptor/ligand bonds that were not reflected in our simulations. For instance, the simulations did not consider that HDMECs presented two adhesion receptors, namely CD36 and ICAM-1^21^, on their surface and that the parasites expressed several PfEMP1 encoding *var* genes at the population level (Supplementary Fig. 2). Furthermore, each of the expressed PfEMP1 molecules is expected to have distinct binding kinetics for ICAM-1 and/or CD36^36,37^, which our simulations also did not take into account.

Previous studies have proposed that the sickle cell trait protects carriers from severe malaria by causing fewer inflammatory reactions and vaso-occlusive events in the microvasculature due to impaired adherence of the corresponding infected erythrocytes to the endothelium^2,4,38^. Our study now shows that the differential firm and dynamic cytoadherence behavior of parasitized HbAS erythrocytes leads to a reduced endothelial cell activation. As a common denominator of endothelial cell activation by cytoadhering *P. falciparum*-infected erythrocytes we monitored nuclear translocation of the NF-κB subunit p65^17^. We explain the reduced endothelial cell activation by two effects: the lower number of firmly cytoadhering parasitized HbAS erythrocytes and the smaller contact footprint generated by dynamically adhering cells. This conclusion is consistent with previous findings demonstrating that endothelial cells can be activated by cell-to-cell contact^33^, depending on the contact time and contact force^18^. Given that activated endothelial cells upregulate surface receptors, thereby contributing to the sequestration of parasitized erythrocytes and, hence, vascular obstruction^17–20^, reduced endothelial cell activation might, therefore, add to the mechanism by which sickle haemoglobin protects carriers from severe malaria.

## Methods

### Blood collection and genotyping

Whole blood was collected by venipuncture into citrate tubes and centrifuged at 800×*g* for 10 min at RT. After discarding the plasma and buffy coat, remaining erythrocytes were washed with RPMI-1640 medium supplemented with 2 mM L‐glutamine and 25 mM HEPES. Packed erythrocytes were stored at 4 °C and used within 2 weeks after collection. The haemoglobin type was determined by cellulose acetate electrophoresis^39^.

### Endothelial cell culture

HDMECs were purchased from PromoCell and cultured in endothelial cell growth medium MV 2 at 37 °C and 5% CO_2_, as recommended by the manufacturer. HDMEC were used between passage 4 and 7.

### *Plasmodium falciparum* culture

The *P. falciparum* FCR3 (Gambia) strain was cultured using wild type and HbAS erythrocytes, as described^40^. Briefly, cultures were maintained at a hematocrit of 3.5% and a parasitemia of not higher than 5% in RMPI-1640 medium supplemented with 2 mM L-glutamine, 25 mM HEPES, 100 µM hypoxanthine, 20 µg ml^−1^ gentamicin and 10% (v/v) heat-inactivated human AB^+^ serum. Cultures were incubated at 37 °C under controlled atmospheric conditions of 3% CO_2_, 5% O_2_ and 92% N_2_ and 96% humidity. Cultures were synchronized, using the sorbitol lysis method^41^, and infected erythrocytes were selected for the presence of knobs, using the gelatin flotation method^42^. Parasites were selected for binding to HDMEC, as described^43^. The knobless *P. falciparum* line was derived from the FCR3 strain by a chromosomal breakage and healing event within the knob-defining *kahrp* gene, resulting in a truncated non-functional *kahrp* gene and the loss of ~100 kbp of DNA from the affected chromosome 2^44^.

### Parasite multiplication rate and multiple infectivity

The parasitized erythrocyte multiplication rate (PEMR) was determined, as described^45^. Briefly, tightly synchronized late-stage infected erythrocytes were enriched by magnetic cell sorting. Concentrated infected erythrocytes (≤98%) were returned to culture, with the parasitemia (0.05–1.0%) being adjusted using the appropriate amount of either fresh HbAA or HbAS erythrocytes. The PEMR was calculated by dividing the final parasitemia at the ring stage by the starting parasitemia at the schizont stage. At least 1000 erythrocytes were analyzed per blood smear. The parasite multiple infectivity (PMI, infected red blood cell containing more than one parasite) was determined from a total of 300 infected red blood cells. Assays were repeated at least three times, with blood obtained from at least two different donors.

### *Var* gene transcriptional profile

The *var* gene transcriptional profile was determined, as described^46^. Briefly, total RNA was extracted from tightly synchronized parasite cultures at the ring stage (~10 h post-invasion), using TRIzol (Invitrogen) as recommended by the manufacturer. Contaminating DNA was removed by DNAse treatment, using the TURBO DNase I (Ambion). RNA was then reverse transcribed to cDNA, using random hexamers and the SuperScript III First-Strand Synthesis System (Invitrogen). A total of 5 µg of starting RNA was sufficient to probe for all genomic *var* genes. The quantitative real-time PCR was performed in 20 µl, using Universal SYBR Green Supermix (BioRad), specific primer pairs for each IT4 *var* gene^47^ and the prepared cDNA. The reactions were assessed on a CFX96 thermocycler (BioRad). The relative transcription was determined by normalization with the housekeeping control gene seryl-tRNA synthetase [PlasmoDB: PF07_0073].

### Flow chamber assay

Flow chamber experiments were carried out, as described^14^. Briefly, HDMEC were grown to confluence under static conditions on parallel plate flow chambers (µ-slide VI^0.4^, Ibidi) pre-coated with fibronectin (10 µg ml^−1^ in PBS). Confluent endothelial cells were washed twice with binding medium (RPMI 1640 supplemented with 2 mM glutamine, 25 mM HEPES, and 0.1% (v/v) BSA, pH 7.2 adjusted with NaOH) and mounted on a stage of an inverted microscope. Flow rates were controlled, using the Harvard Apparatus syringe pump after attaching it to the flow chamber inlet via silicon tubing. For flow chamber experiments, magnet purified infected erythrocytes were labeled using 0.4 µl ml^−1^ SYBR Green for 1 h at 37 °C. The number of cells was counted, using a cell counter (Beckman Coulter), and adjusted to 1 × 10^6^ infected cells ml^−1^ in binding medium. A total of 5 ml of cell suspension, pre-warmed at 37 °C, was perfused over the confluent HDMEC monolayer in the microfluidic chamber at different wall shear stresses, ranging from 0.03 to 0.2 Pa. For each condition, the SYBR Green fluorescence signals were recorded (time interval of 0.033 s) in different fields along the center line of the chamber, using a Zeiss inverted microscope (EGFP HC Filter set Ex/Em: 456–490 nm/500–540 nm, Axio Observer, 40× magnification) (Carl Zeiss). Following perfusion with the cell suspension, unbound red blood cells were washed out for 5 min using binding medium and the corresponding wall shear stress. At least 6 randomly chosen fields of view were analyzed for cytoadhering red blood cells. Fluorescence intensity amplitude and translational velocity were obtained for individual cells post-video analysis, using the TrackMate plugin from ImageJ (v2.7.3). Trophozoites (26 ± 6 h post-invasion) and schizonts (36 ± 6 h post-invasion) infected HbAA and HbAS erythrocytes were compared. Parasite developmental stage was monitored by Giemsa-stained thin blood smears. Where indicated, cytoadhesion blocking antibodies directed against CD36 (FA6-152, Abcam) and ICAM-1 (CD54, 15.2, Serotec) were added (4 µg ml^−1^ in HDMEC culture media) for 1 h under regular culture conditions prior to the flow chamber experiments.

### Quantification of cytoadhesive phenotypes

From the recorded fluorescence signals, two parameters were derived on a single cell basis: the fluorescence intensity and the translational velocity. The fluorescence intensity signals were normalized to the minimum intensity value for comparative reasons and parameterized to yield the following quantitative readouts: fluorescence amplitude difference, contact time, mean translational velocity, and the Pearson correlation coefficient between the fluorescence and the velocity trajectories^14^. Maximum and minimum amplitude values were identified, using a peak detection algorithm provided by the Python software. The amplitude difference, Δ*a* = 〈*a*_max_ − *a*_min_〉, was subsequently computed by measuring the difference between the average maximum amplitude and the average minimum amplitude. The amplitude difference was given in arbitrary units. To determine the contact time, a cutoff value *δ* = 0.05 arbitrary units was chosen and the contact time was estimated as the time taken by the cell to go from 〈*a*_max_〉 − *δ* to 〈*a*_max_〉 and back to 〈*a*_max_〉 − *δ*. Due to the peak character of the region of interest, the exact value of the cutoff value had little effect on the outcome of the results. A negative Pearson correlation coefficient indicated a strong anti-correlation between the fluorescence amplitude and the translation velocity. We further measured the dwell time as the inverse of the angular velocity^36^. To measure the angular velocity from fluorescence amplitude profiles, we took advantage of the fact that there is an angular change of *π*/2 between two consecutive maximum and minimum amplitude points in a fluorescence amplitude trajectory. The amplitude difference d*a* was translated to d*θ* and the angular velocity was computed and, hence, the dwell time. In this way, dwell time was roughly estimated at every angle from *θ* = 0 to *θ* = 2*π* throughout one rotational cycle and then averaged over many amplitude trajectories.

### Endothelial cell activation assay and NF-ĸB translocation

Immuno-localization of NF-ĸB in HDMEC was evaluated by immunofluorescence confocal microscopy after co-culturing the endothelial cells with infected erythrocytes, as described^17^. Briefly, HDMEC were grown to confluence in flow chambers (µ-slide VI^0.4^, Ibidi, Germany) pre-coated with fibronectin (10 µg ml^−1^ in PBS). Infected HbAA and HbAS erythrocytes at the trophozoite stage (26 ± 6 h post-invasion) were magnet-enriched and resuspended in endothelial cell culture medium without hydrocortisone. A total of 7.5 × 10^5^, 3 × 10^6^, 1 × 10^7^, 1.5 × 10^7^ infected erythrocytes were added per flow chamber well (150 µl well volume). HDMEC and infected erythrocytes were subsequently co-cultured under static conditions for 2 h at 37 °C and 5% CO_2_. Afterwards, HDMEC were washed three times with ice-cold PBS before the cells were fixed with 4% (v/v) paraformaldehyde in PBS for 15 min at RT, permeabilized with 0.2% (v/v) Triton X-100 in PBS for 15 min at RT and blocked with 3% (wt/v) BSA in PBS for 30 min at RT. Endothelial cells were incubated with 1:200 rabbit polyclonal IgG anti-human NF-kB p65 subunit (Poly6225, Biolegend) overnight at 4 °C. Following three washes with PBS, Alexa-488 conjugated goat anti-rabbit IgG (ThermoFisher) was added (1:400) for 1 h at RT. Nuclei were counterstained with 5 µM Hoechst for 10 min at RT. Cells were imaged immediately, using a Zeiss confocal microscope (LSM510, Carl Zeiss). Hoechst (Ex/Em 351 nm/385–470 nm), Alexa-488 (Ex/Em 488 nm/505–550 nm) and DIC images were acquired. The following microscope settings were used for the image acquisition: pinhole of 1.71 airy units, scan speed of 1.6 µs/pixel, frame averaging of 2. Photomultiplier tube gain and offset were adjusted to obtain sub-saturating fluorescence intensity with an optimal signal-to-noise ratio. Digital images were recorded from at least 6 random fields of view (63×/1.2 NA W objective) for each sample. The nuclear labeling index (NLI), defined as the percentage of positive p65-NF-ĸB immunolabeled nuclei from the total number of nuclei observed by Hoechst staining, was calculated, as described^48^, and expressed as mean ± SEM from at least three biological replicates. Where indicated, NF-ĸB nuclear translocation was assessed, using infected HbAA and HbAS erythrocytes pre-incubated with 50 µg ml^−1^ of purified ICAM-1 (ACRO Biosystems) and/or CD36 protein (Sino Biological) for 1 h at 37 °C. In addition, an isogenic knobless FCR3 line was investigated (see above for further details). Uninfected HbAA and HbAS erythrocytes served as negative controls and TNFα (100 units ml^−1^) served as a positive control. For flow experiments, a total of 1 × 10^8^ infected erythrocytes resuspended in 5 ml medium were perfused at 0.03 Pa over the confluent HDMEC. Unbound cells were subsequently washed out by perfusing the chamber for 5 min with medium at the appropriate hydrodynamic condition. The chamber was then incubated for 2 h under regular endothelial cell culture conditions and NF-ĸB was assessed as described above.

### IL-6 secretion and ICAM-1 surface expression

As indicators of endothelial cell activation, IL-6 secretion and ICAM-1 surface expression were monitored as described^33^. HDMEC between passage 4 and 7 were grown to confluence in 12-well plates. Confluent HDMEC were co-cultured for 16 h with 5 × 10^6^ magnet-enriched trophozoite-infected erythrocytes, resuspended in 1 ml endothelial cell culture medium without hydrocortisone. TNF-α (100 units ml^−1^) served as a positive control for activation, whereas uninfected erythrocytes and medium were used as negative controls. After the incubation time, the supernatant was collected for IL-6 detection by ELISA (human IL-6 ELISA MAX^TM^ Deluxe set, Biolegend). ICAM-1 surface expression on endothelial cells was quantified by flow cytometry. Briefly, endothelial cells were detached with trypsin-EDTA and collected by centrifugation at 300×*g* for 5 min at RT. After washing the cell pellet with ice-cold FACS buffer (PBS, 2% (v/v) fetal calf serum (FCS) and 0.1% (w/v) sodium azide), 2–10 × 10^5^ cells were stained with 1:50 PE-labeled monoclonal mouse anti-human ICAM-1 (Biolegend) in 50 µl FACS buffer for 30 min on ice in the dark. Cells were washed and fixed with 2% (v/v) formaldehyde in PBS. Fluorescence intensity signals (Ex/Em 488 nm/575 nm) and forward scatter light signals were recorded from a total of 10,000 cells, using the FACS Calibur (Becton and Dickinson). Data analysis was performed, using the CellQuest Pro software (Becton and Dickinson).

### AFM imaging

Knob shape and density were determined, as described^30^. From a synchronized late-stage-dominated *P. falciparum* parasite culture, 1 ml was collected. The cell suspension was stained with 2% (v/v) filtered Giemsa solution for 10 min before being centrifuged at 500×*g* for 3 min. 3 μl of pellet was used to make a thin blood smear on a clean glass slide. The smear was air-dried. A grid was glued to the back of the glass slide and infected erythrocytes were identified by microscopy and their location on the grid was noted for subsequent AFM probing. AFM images were investigated using the JPK Nano Wizard III (JPK Instruments AG) mounted on an inverted microscope. Images for knob analysis were captured in air under ambient conditions with the tapping mode using a silicon cantilever (PPP-NCLR, Nanosensors) with a spring constant of 21–98 N/m and a resonant frequency of 160 kHz. The images were 256 × 256 pixels and captured at a scan speed of 0.5–1.0 Hz depending on the scan size, which ranged from 1 to 10 μm. In order to minimize the noise, integral and proportional gains as well as scan speed were optimized individually. Using the JPK processing software, identification of knobs was achieved by comparing the amplitude image and the height image. Number of knobs are given as density per µm^2^. From the knob height and diameter values, the knob surface was calculated using the equation of a spherical cap:

$${\mathrm{spherical}} \;{\mathrm{cap}} = \pi (a^2 + h^2)$$

where *a* is the knob radius and the *h* is the knob height. Outliers were removed by the jackknife method.

### Flicker spectroscopy

Uninfected and infected erythrocytes (trophozoite-stage) were resuspended in RPMI-1640 medium supplemented with 0.1% (v/v) BSA (pH 7.4) at a hematocrit of ~0.1%. Cells were incubated for 30 min at 37 °C in Petri dishes with RCA cleaned glass bottom. Videos were recorded on a Zeiss microscope at a rate of 40 fps (Apo 100× 1.4 NA oil immersion objective) using an Orca Flash4.0 CMOS camera (Hamamatsu). Data analysis was carried out using a self-written script in Matlab (Mathworks, Matlab r2016b). First the closed contour of the cell was segmented in the 2D image plane of the cell equator. Following the standard approach^49,50^, the fluctuation amplitude *h*(*θ*_*i*,_*t*_*n*_) at discrete angle *θ*_*i*_, and discrete time *t*_*n*_ is determined from the deviation of the contour from its mean position. This quantity is then transformed to Fourier space and averaged. Bending modulus (*κ*), tension (*σ*), and confinement parameter (*γ*) were obtained from a fit to the power spectrum of the Helfrich-model for planar membranes in harmonic confinement as a function of *q*_*x*_ with the dependence on *q*_*y*_ projected onto the equator in real space^51,52^:

$$\langle h\left( {q_x,y = 0} \right)^2\rangle = \frac{{k_{\mathrm B}T}}{L}\sqrt {\frac{\kappa }{{2\left( {\sigma ^2 - 4{\mathrm{\kappa }}\gamma } \right)}}} \\ \left[ {\frac{1}{{\sqrt {2\kappa q_x^2 + \sigma - \sqrt {\sigma ^2 - 4\kappa \gamma } } }} - \frac{1}{{\sqrt {2\kappa q_x^2 + \sigma + \sqrt {\sigma ^2 - 4\kappa \gamma } } }}} \right]$$

where *k*_B_ is the Boltzmann constant, *T* is temperature, and *L* is the circumference of the associated circular contour. The power spectrum at low *q*_*x*_ is dominated by *γ* and *σ*, and by *κ* at high *q*_*x*_.

### Mesoscopic computer simulations of cells in shear flow

In order to model the movement of infected erythrocytes in shear flow, which in general has a complex shape and therefore a complex hydrodynamic flow field around it, two model inputs were required: a deformable cell model and an explicit solvent method. The deformable red blood cell model was adapted from Fedosov et al.^26^ and the solvent was implemented with Multiparticle Collision Dynamics (MPCD)^27,53^. In contrast to other mesoscopic simulation methods such as Dissipative Particle Dynamics (DPD) or the Lattice Boltzmann Method (LBM), MPCD is relatively easy to implement for problems involving extended deforming surfaces in flow^54^. It consists of solvent particles with mass *m*, sorted into a cubic mesh with side length *a*. *m* and *a* set the basic mass and length scales in the system. The solvent particles are propagated in time in two steps, namely streaming and collision. For streaming we simply have:

$$r_i\left( {t + \Delta t_{\rm cd}} \right) = r_i\left( r \right) + {\mathbf{v}}_{\mathbf{i}}\left( t \right)\Delta t_{\rm cd}$$

where Δ*t*_cd_ is the collision time step and ***v***_**i**_(*t*) is the velocity of particle *i*. The collision step includes momentum exchange among solvent particles and also includes solute particles, which in our case are the vertices of the cell surface:

$${\mathbf{v}}_{\mathbf{i}}\left( {t + \Delta t_{\rm cd}} \right) = {\mathbf{v}}_{{\mathbf{cm}}}\left( t \right) + {\Bbb R}(\alpha )({\mathbf{v}}_{\mathbf{i}}\left( t \right) - {\mathbf{v}}_{{\mathbf{cm}}}\left( t \right))$$

where ***v***_**cm**_(*t*) is center of mass velocity of the computational box to which particle *i* belongs. ℝ(*α*) is the stochastic rotation matrix. It is generated by choosing a random axis for each computational box. The angle of rotation *α* is fixed throughout the simulation. The choices for rotation angle *α*, time step Δ*t*_cd_ and the density of solvent particles per cell determines the viscosity of the fluid^55^ and this can be checked in model simulations, e.g., of Poiseuille flow.

The shape of the red blood was modeled using a 2D triangular meshwork with the vertices connected by springs. The positions of these vertices are given by $\{ \vec r_i\}$. The total number of edges and triangles are *N*_e_ and *N*_t_, respectively. The potential energy of the system is:

1 $$V(\{ x_i\} ) = V_{\mathrm{in-plane}} + V_{\mathrm{bend}} + V_{\mathrm{area}} + V_{\mathrm{volume}}$$

The first term is the in-plane elastic energy of the network:

$$V_{\mathrm{in-plane}} = \mathop {\sum }\limits_{j = 1}^{N_{\mathrm e}} \frac{{k_{\mathrm B}Tl_{\mathrm m}\left( {3x_j^2 - 2x_j^3} \right)}}{{4p\left( {1 - x_j} \right)}} + \frac{{k_p}}{{\left( {n - 1} \right)l_j^{n - 1}}}$$

where the first term is the attractive worm-like chain (WLC) potential and the second term is a short-ranged repulsive potential that could result from steric interactions. Together these two potentials give rise to spring-like potential with non-zero edge length. In the first term, *p* is persistence length, *l*_m_ is maximum extension of the edge and *x*_*i*_ = *l*_*i*_/*l*_m_. In the second term, *k*_*p*_ is the force coefficient and *n* is an exponent which was chosen to be 2 in simulations. The second term in Eq. (1) represents the bending energy of the triangular mesh:

$$V_{\mathrm{bend}} = \mathop {\sum }\limits_{i = 1}^{N_{\mathrm e}} \kappa _{\mathrm b}(1 - \mathrm{cos}(\theta _i - \theta _0)) \approx \mathop {\sum }\limits_{i = 1}^{N_{\mathrm e}} \frac{{\kappa _{\mathrm b}}}{2}\left( {\theta _i - \theta _0} \right)^2$$

where *κ*_b_ is the bending modulus, *θ*_*i*_ is the angle between two consecutive triangles having common edge *i*, and *θ*_0_ is the preferred angle. The bending moduli *κ*_b_ is related to the bending rigidity in the Helfrich–Hamiltonian by $\kappa _{\mathrm b} = \left( {\frac{2}{{\sqrt 3 }}} \right)\kappa _{\mathrm c}$. The last two terms in Eq. (1) constrain surface area and volume of the red blood cell, respectively:

$$V_{\mathrm{area}} = \frac{{k_{\mathrm a}\left( {A - A_0} \right)^2}}{{2A_0}} + \mathop {\sum }\limits_j^{N_{\mathrm t}} \frac{{k_{\mathrm d}\left( {A_j - A_j^0} \right)^2}}{{2A_j^0}}$$

$$V_{\mathrm{volume}} = \frac{{k_{\mathrm v}\left( {V - V_0} \right)^2}}{{2V_0}}$$

where *k*_a_ and *k*_d_ are the global area and local area constrain coefficients, respectively, and *k*_v_ is the global volume constrain coefficient. The elastic moduli of the membrane such as shear modulus *μ*_0_, Young’s modulus *Y*, and Poisson ratio *ν* can be derived in terms of the microscopic model parameters for a hexagonal mesh:

$$\mu _0 = \frac{{\sqrt 3 k_{\mathrm B}T}}{{4pl_{\mathrm m}x_0}}\left( {\frac{{x_0}}{{2\left( {1 - x_0} \right)^3}} - \frac{1}{{4\left( {1 - x_0} \right)^2}} + \frac{1}{4}} \right) + \frac{{\sqrt 3 k_p\left( {n + 1} \right)}}{{4l_0^{n + 1}}}$$

$$Y = \frac{{2K\mu _0}}{{K + \mu _0}}$$

$$\nu = \frac{{K - \mu _0}}{{K + \mu _0}}$$

where *K* = 2*μ*_0_ + *k*_a_ + *k*_d_ is the area compression modulus, *l*_0_ is the equilibrium edge length, *l*_m_ is the maximum edge length, and *x*_0_ = *l*_m_/*l*_0_. For high values of *k*_a_ and *k*_d_, the membrane becomes nearly in-compressible. Then the Young’s modulus becomes close to 4*μ*_0_ and the 2D Poisson ratio close to 1, the maximal possible value. In our simulations, a Poisson ratio around 0.96 was used. The membrane was coarse-grained into *N* = 600 vertices, which sets the edge length close to 500 nm as the red blood cell has a typical size of 8 μm. The effective persistence length was set close to 1 nm. We use a stress-free model as proposed in refs. ^56,57^, i.e., instead of choosing a global equilibrium edge length, the value of *k*_*p*_ was computed for a given local edge length (which will be the equilibrium length for that edge throughout our simulations) for a given shear modulus of the membrane. In this way, each edge has its own equilibrium length which is fixed with the initial configuration, which here in turn is taken from experiments.

The number of knobs *m* from *N* vertices was chosen as follows: the ratio of each triangle area and the total surface area of all faces or triangles were computed and the total sum of these ratios was set as 1. The selection of vertices to represent the knobs was done by generating a random number (0, 1) and calculating the cumulative sum of the area ratios until it crossed the chosen random number. The available vertex from the latest triangle in the cumulative sum was selected as a knob. This procedure was performed until the *m* number of knobs was chosen.

The membrane vertices interact with the solvent particles not only through the collision step, but also through the rule that the solvent particles inside and outside the membrane are separated. To achieve this, during every time step each solvent particle goes through bounce-back reflections with the nearby triangular plaquettes. The membrane dynamics is integrated using the velocity Verlet-algorithm with time step Δ*t*_md_ < Δ*t*_cd_. The mass of membrane vertex *M* is chosen to be 10 m (as is the mass of the rigid parasite). The MPCD time step Δ*t*_cd_ is chosen to be 0.02 and Δ*t*_md_ is 0.0025. The solvent particle number in each collision box is 10 and the angle *α* is fixed to 3*π*/4. The simulation box size is 35 μm × 25 μm × 25 μm and total number of solvent particles are close to 2 × 10^6^. In the current simulations, we chose internal and external viscosity to be the same, as done before for MPCD-simulations for red blood cells^58^. All the parameters in the system are expressed in MPCD units, which are dimensionless. To match the simulation parameters qualitatively with experimental values, we scaled simulation units to experimental units following the procedure described in Fedosov et al.^56,57^. This scaling procedure sets basic length scale *a* is roughly equivalent to 500 nm and *k*_B_*T* in model units is 1 *k*_B_*T* in physical units. The time scaling from model units to physical units is done by equating characteristic red blood cell relaxation time $\sim \eta D_0^3/\kappa _{\mathrm c} \sim 1.7{\mathrm{s}}$ for *η* ∼ 1mPa s. The effective Reynolds number of our simulations is Re ∼ 0.7, as it is typical for MPCD-simulations in the low-Reynolds number regime (smaller values are prohibited by the high computational cost of using even more solvent particles).

Finally, adhesive bond dynamics between the cell membrane and substrate was implemented, similar to Brownian dynamics of infected erythrocytes in shear flow^14^. Ligands were arranged uniformly on the substrate with spacing *δx* = *δy* = 250 nm. Receptors were placed at the vertex positions. At each time step Δ*t*_md_, possible bond association and dissociation events were allowed. For bond association, each receptor bonds to the nearest available ligand on the substrate when it is less than a critical distance *r*_0_ = 250 nm away, with a constant on-rate *κ*_on_. For bond dissociation, we use Bell’s model^59^, i.e., off-rate *κ*_off_ depends on the force through $\kappa _{\mathrm{off}} = \kappa _{\mathrm{off}}^0{\mathrm e}^{\left( {\frac{F}{{F_{\mathrm d}}}} \right)}$, where the internal force scale *F*_d_ is assumed to be roughly 10 pN. The receptor–ligand bond is modeled with a cable force model^14,60^: *F*(*l*) = *k*_s_(*l* − *l*_0_) when *l* is greater than *l*_0_, where *k*_*s*_ ≃ 0.1 pN/nm and *l*_0_ = 50 nm. Otherwise it vanishes because a cable does not resist compression. At each step, bond association occurs with probability $P_{\mathrm{on}} = 1 - \exp ( - \kappa _{\mathrm{on}}\Delta t_{\rm md})$ and bond dissociation with $P_{\mathrm{off}} = 1 - \exp ( - \kappa _{\mathrm{off}}\Delta t_{\rm md})$.

Similar to the experiments, we measured as main readouts the center of mass velocity of the cell ${\vec{\mathbf v}}_{{\mathbf{cm}}}$ and the fluorescence amplitude *I*(*t*) defined as follows:

$${\vec{\mathbf v}}_{{\mathbf{cm}}}\left( t \right) = \frac{{\vec r_{\rm cm}\left( t \right) - \vec r_{\rm cm}\left( {t - \Delta t} \right)}}{{\Delta t}}$$

$$I( t ) = \exp ( - \frac{{\left( {z_{cm}(t) - z_0} \right)^2}}{{2\sigma ^2}})$$

where *z*_cm_(*t*) is the *z*-component of the center of mass of the cell at time *t*, *z*_0_ is the initial reference height and *σ* ≃ 1.2 μm is the standard deviation. The flow lines in Fig. 3a are generated by averaging solvent particle velocities with bin width of 3*a* in each direction and the fluid flow is only shown around the cell rather than full simulation box.

### Statistical analysis

Statistical analyses were performed using the Sigma Plot (v.13, Systat) software. Statistical significance was determined using the two-tailed Student's *t*-test, one-way ANOVA, one-way ANOVA on ranks or the *F*-test, where appropriate.

### Ethics approval

This study was approved by the ethics review board of Heidelberg University and Mannheim University. Written informed consent was obtained from all volunteers before blood sample collection.

### Code availability

The codes and algorithms used in this study are available at https://github.com/Dasanna/iRBC-code.

## Electronic supplementary material

Supplementary Information

Supplementary Data 1

Description of Additional Supplementary Files

Supplementary Movie 1

Supplementary Movie 2

Supplementary Movie 3

Supplementary Movie 4

Supplementary Movie 5

Supplementary Movie 6

Supplementary Movie 9

Supplementary Movie 10

Supplementary Movie 11

Supplementary Movie 12

Supplementary Movie 7

Supplementary Movie 8

## Data Availability

The authors declare that the data supporting the findings of this study are available within the Article and its Supplementary Information files, or are available from the authors upon request. The original data underlying this article are compiled in Supplementary Data 1 (Excel file) and are available via the Dryad Digital Repository at 10.5061/dryad.45sp7nr.
