# Supplementary material for: The sickle cell trait affects contact dynamics and endothelial cell activation in Plasmodium falciparum-infected erythrocytes
Source: Commun Biol. 2018 Nov 30;1:211. doi: 10.1038/s42003-018-0223-3 (PMC6269544; doi:10.1038/s42003-018-0223-3)
Supplement: Supplementary file 1 — Supplementary Information [file 42003_2018_223_MOESM1_ESM.pdf]

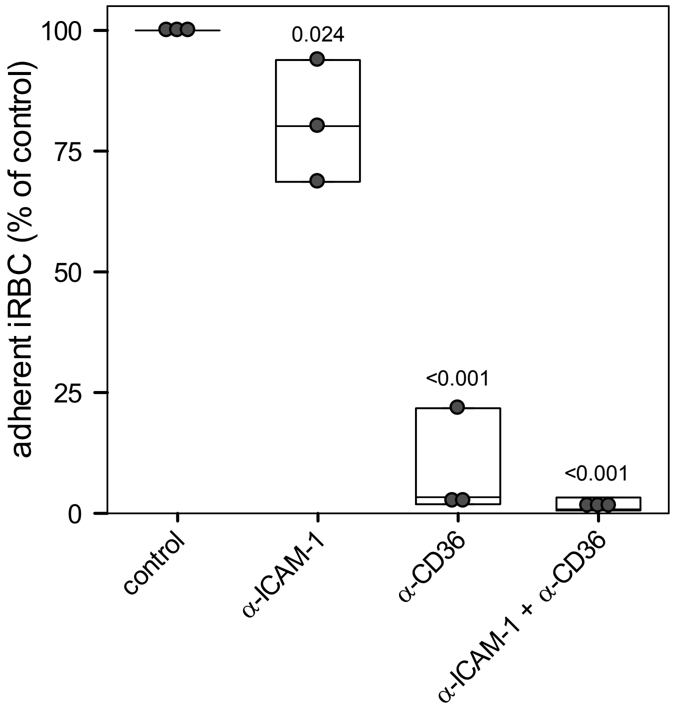

Supplementary Fig. 1 Cytoadhesion of parasitized erythrocytes to ICAM-1 and CD36 exposed on human dermal microvascular endothelial cells (HDMEC). Percentage of parasitized erythrocytes remaining adherent on HDMEC at a wall shear stress of 0.05 Pa after blocking CD36 and/or ICAM-1 binding with specific monoclonal antibodies. Values were normalized to controls. A box plot analysis is overlaid over the individual data points, with the median, 25% and 75% quartile ranges shown for three independent biological replicates. Statistical significance was determined using Holm-Sidak one-way ANOVA.

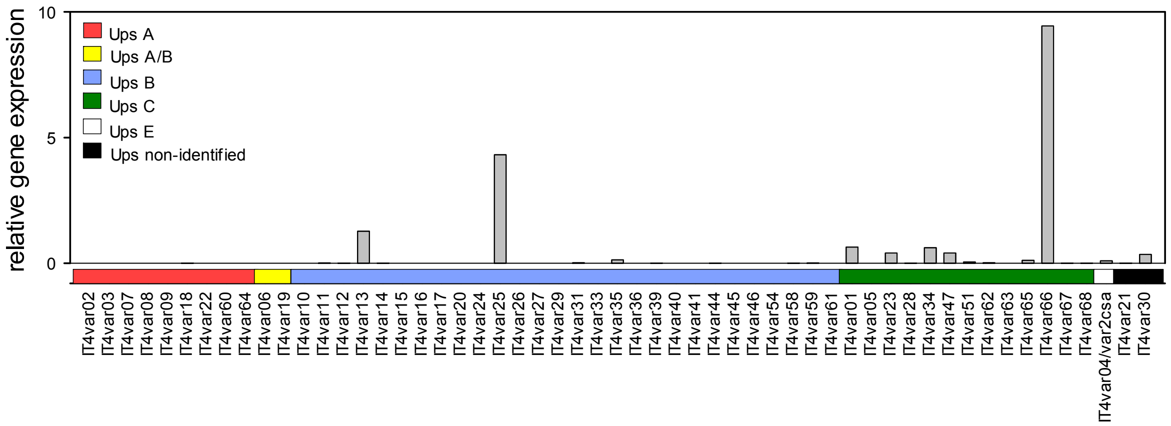

Supplementary Fig. 2 Characterization of *var* genes expressed by FCR3^{HDMEC} at the population level. Transcription of *var* genes was assessed after repeated panning of the *P. falciparum* strain FCR3 infected erythrocytes over HDMEC. Results were normalized to the housekeeping control gene *seryl synthetase*. Genes are organized based on the upstream sequence (Ups).

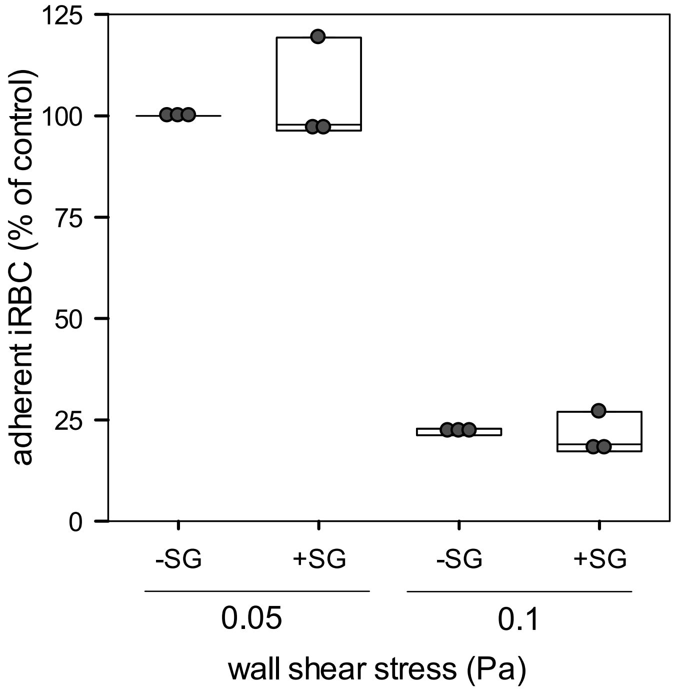

Supplementary Fig. 3 Effect of SYBR Green staining on the cytoadhesion of *P. falciparum*-infected erythrocytes to HDMEC. Infected HbAA erythrocytes were stained with SYBR Green (+SG) or left unstained (-SG), and the number of cells cytoadhering to HDMECs was determined and normalized to the control value in the absence of SYBR Green. The experiment was performed in flow chambers with wall shear stresses of 0.05 Pa and 0.1 Pa. A box plot analysis is overlaid over the individual data points, with the median, 25% and 75% quartile ranges shown for three independent biological replicates. The data were analyzed using the Student's two tailed t-test (confidence interval: 95%; p=0.578 for 0.05 Pa and 0.0707 for 0.1 Pa.

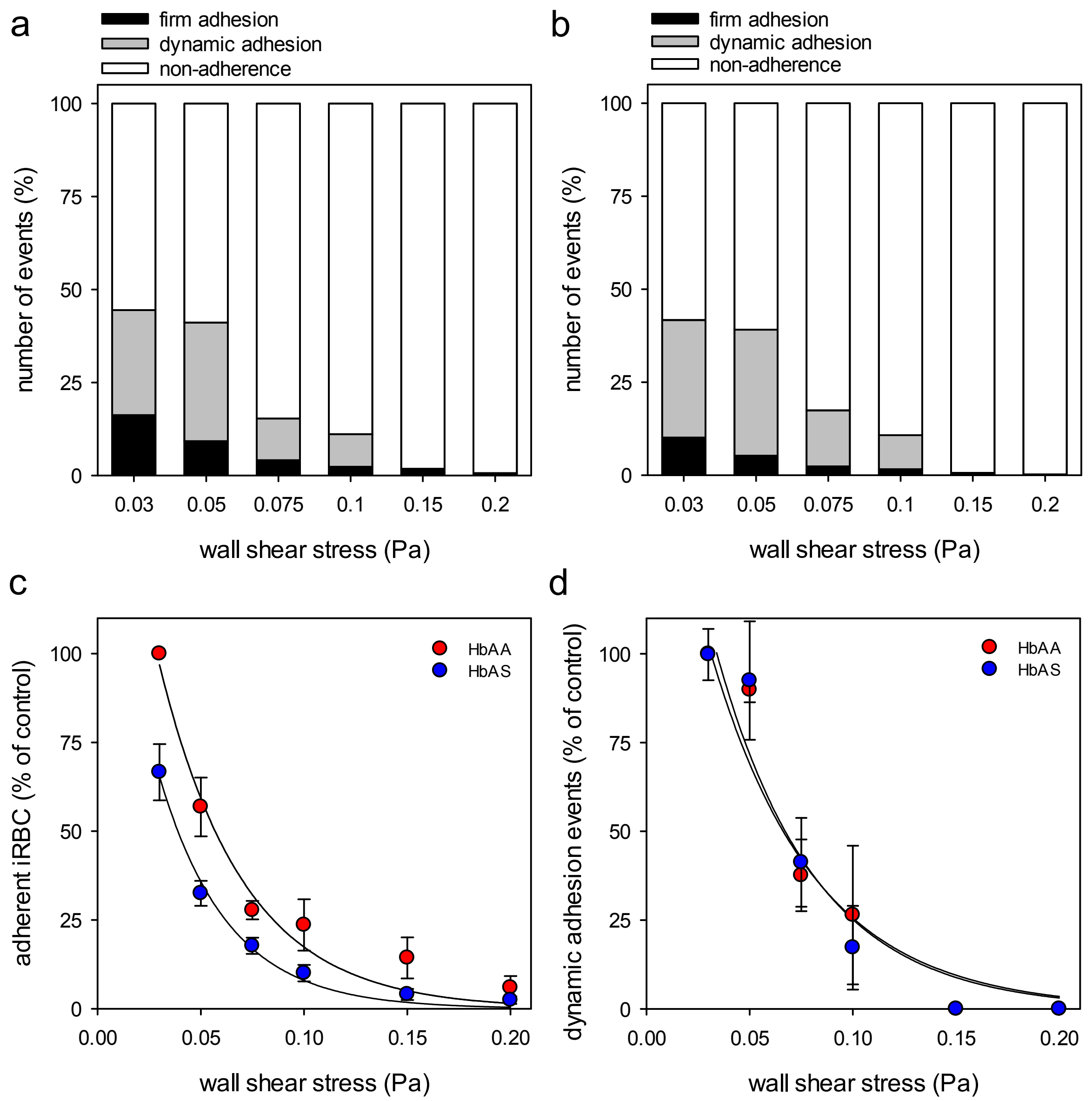

Supplementary Fig. 4 Effect of HbAS on *P. falciparum*-infected erythrocytes adhesion to HDMEC under flow conditions. **a, b** Stacked bar charts representing relative proportions of various categories of motion behaviors observed for infected (**a**) HbAA and (**b**) HbAS erythrocytes ($5x10^6$) superfused over confluent HDMEC at physiological shear stress ranging from 0.03 to 0.2 Pa. The results from at least four independent biological replicates are shown. Note that the relative levels of dynamic and firm adhesion may depend on the parasite strain and the PfEMP1/receptor pair. **c** Percentage of firmly cytoadhering infected HbAA and HbAS erythrocytes as a function of the wall shear stress. The mean ± SEM are shown for at least four independent biological replicates. **d** Percentage of dynamic adhesion events of parasitized HbAA and HbAS erythrocytes to HDMEC. Values are expressed relative to the infected HbAA erythrocytes control group binding at a shear stress of 0.03 Pa. The mean ± SEM are shown for at least four independent biological replicates.

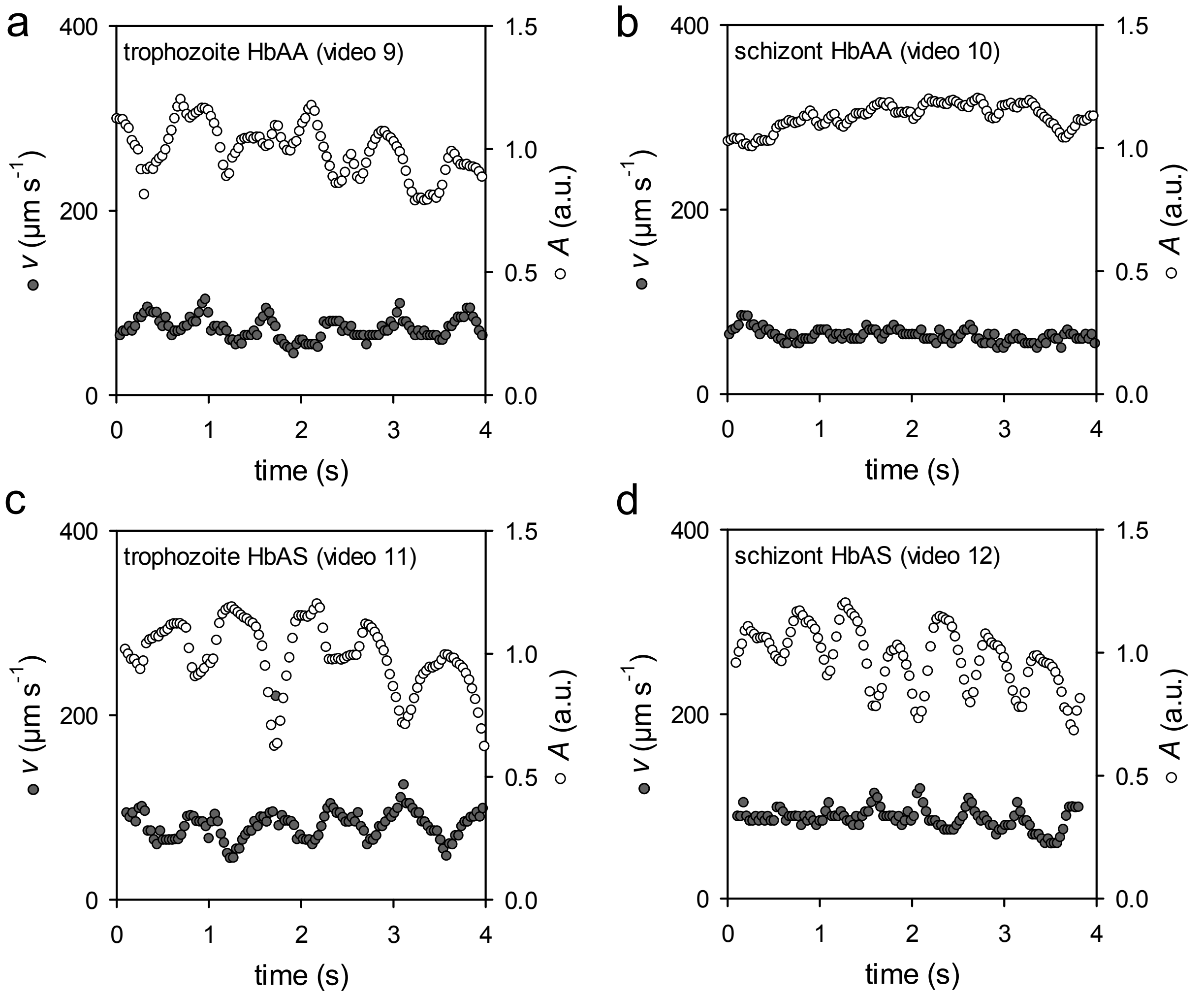

Supplementary Fig. 5 Stage-specific and erythrocyte variant-specific adhesion dynamics. **a-d** Representative trajectories of the translational velocity, v, and fluorescence amplitude, *A*, of infected HbAA and HbAS erythrocytes at the trophozoite and schizont stage. The red arrows indicate velocity peaks, suggestive of a transient detachment of the parasitized HbAS erythrocyte from the substratum. The corresponding movie to the selected trajectories can be found in the supplementary information (Supplementary Movies 9-12). Wall shear stress, 0.03 Pa. a.u., arbitrary unit.

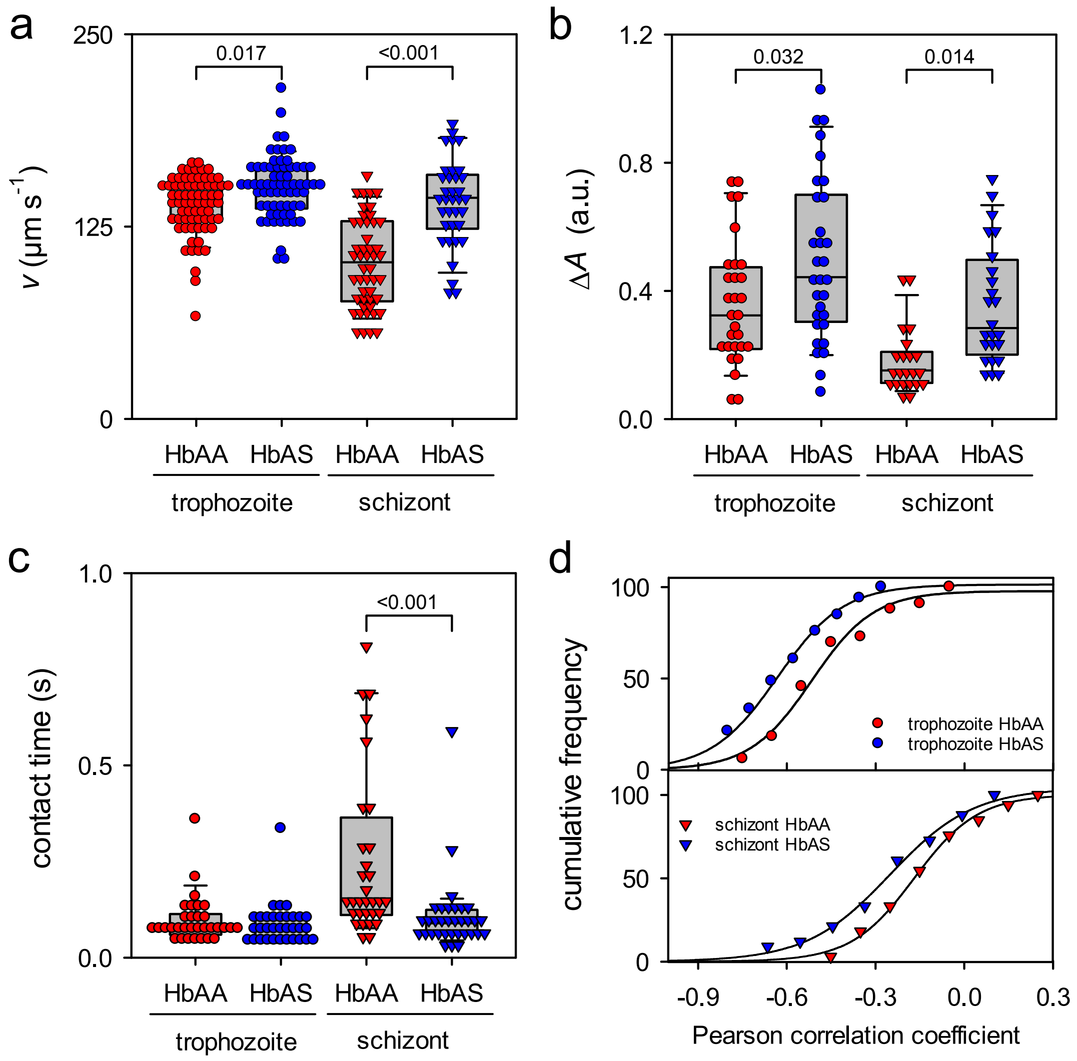

Supplementary Fig. 6 Quantitative analysis of the adhesion dynamics. The individual velocity and amplitude trajectories were parameterized and the following parameters were obtained: (**a**) the mean translational velocity, (**b**) fluorescence intensity amplitude difference, (**c**) contact time and (**d**) Pearson correlation coefficient between fluorescence amplitude and velocity profile. Note that the data for parasitized HbAA and HbAS erythrocytes are statistically different, according to an F-test (trophozoites: F=25, DF=21, p<0.001; schizonts: F=9, DF=21, p<0.001). Each data point corresponds to a single cell measurement, with at least 27 cells being measured per condition. Infected HbAA and HbAS erythrocytes at the trophozoite and schizont stage were compared. A box plot analysis is overlaid over the individual data points, with the median, 25% and 75% quartile ranges and the standard error of the mean being shown. Statistical significance was assessed, using Dunn's ANOVA on ranks. Wall shear stress, 0.05 Pa.

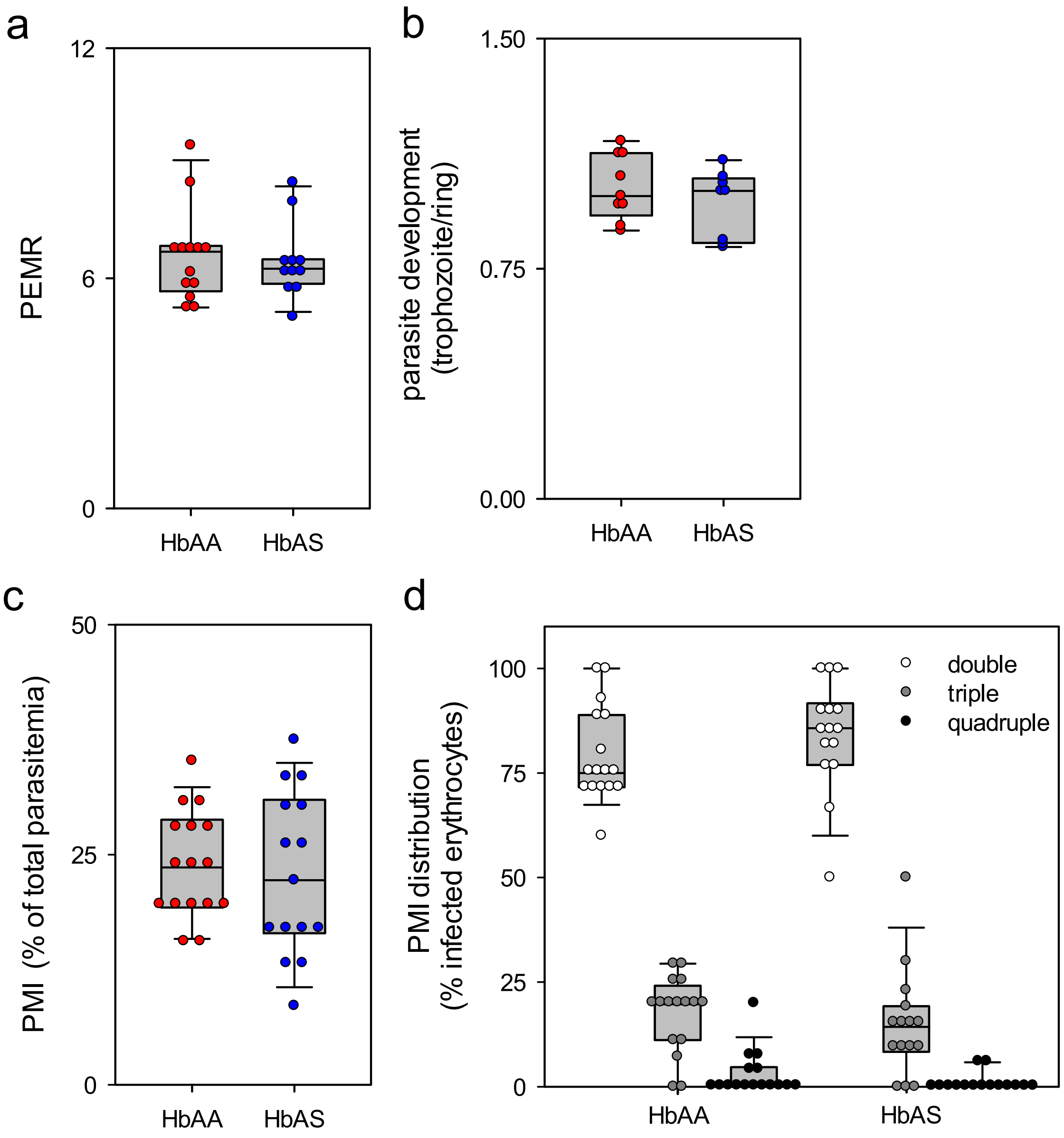

Supplementary Fig. 7 Comparative analysis of parasite growth in HbAA and HbAS erythrocytes. (**a**) Parasitic erythrocyte multiplication rate (PEMR), (**b**) intraerythrocytic parasite development from rings to trophozoites, (**c**) percentage of multiple infectivity (PMI), and (**d**) the distribution of multiple infectivity. A box plot analysis is overlaid over the individual data points, with the median, 25% and 75% quartile ranges shown for three independent biological replicates.

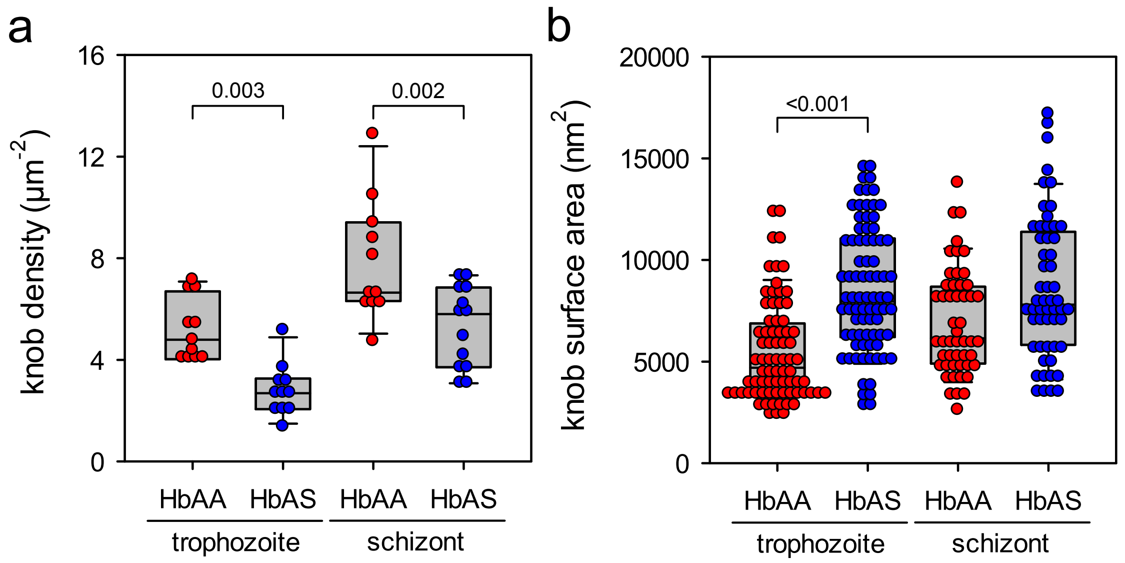

Supplementary Fig. 8 Knob density (**a**) and knob surface area (**b**) *for* HbAA and HbAS erythrocytes infected with the *P. falciparum* strain FCR3^{HDMEC}. Knob density and knob surface area were determined using atomic force microscopy. Each data point represents determinations from single cells. Cells from three independent biological replicates were analyzed. Statistical significance was assessed, using Dunn's ANOVA on ranks.

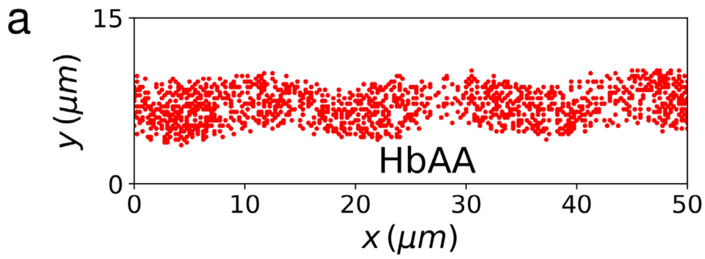

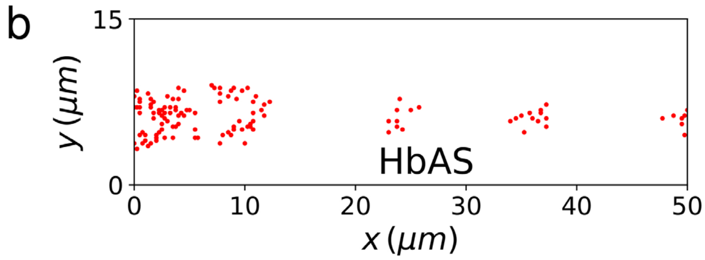

Supplementary Fig. 9 Footprints of parasitized HbAA (**a**) and HbAS erythrocytes (**b**) at the trophozoite stage. Representative examples of simulated contact footprints are shown for infected HbAA and HbAS erythrocytes at the trophozoite at a wall shear stress of 0.2 Pa. Simulation parameters see results section and figure legend 5.

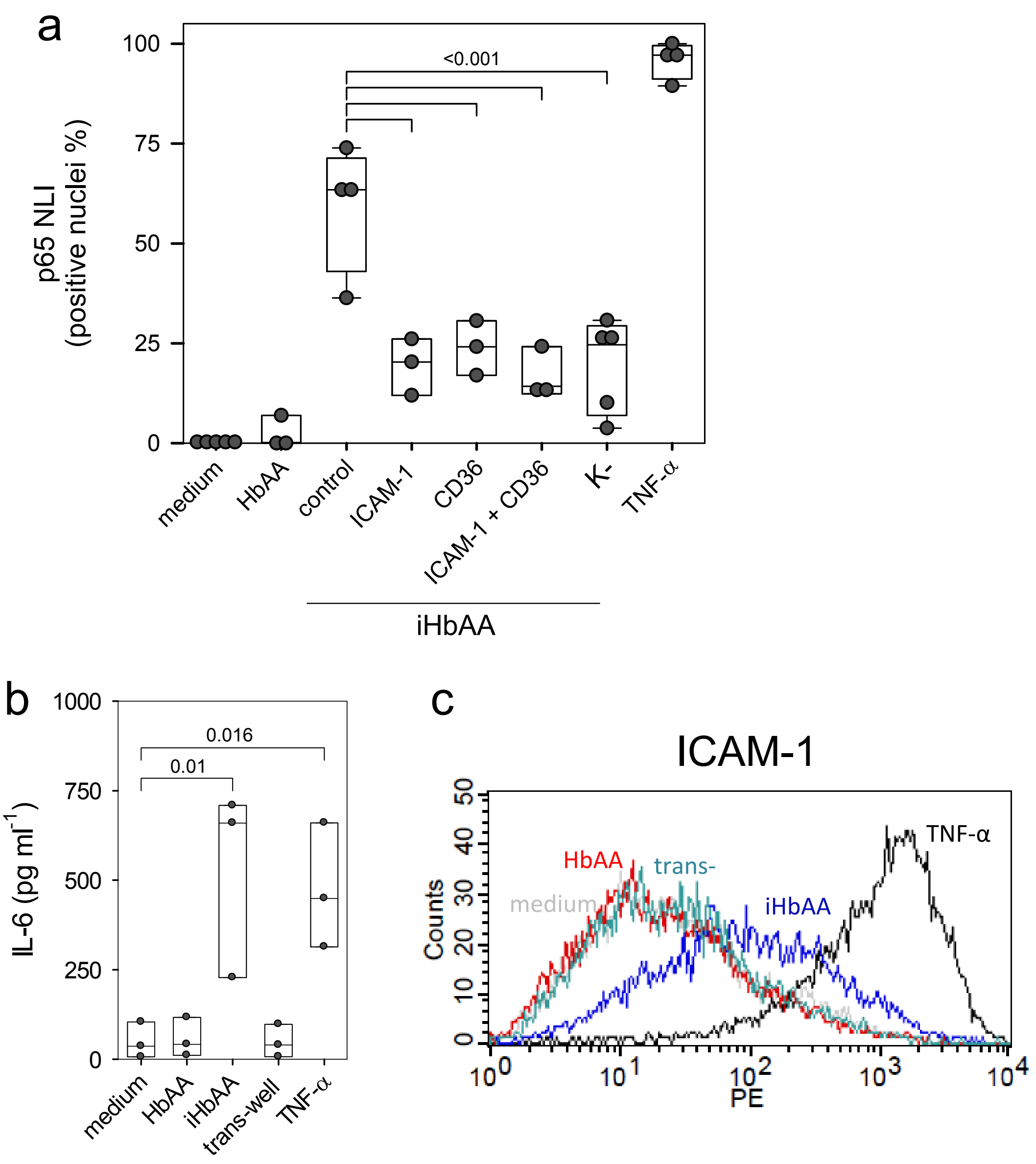

Supplementary Fig. 10 Activation of HDMEC by *P. falciparum*-infected erythrocytes via cell-to-cell contact. **a** The percentage of HDMECs positive for nuclear p65-NFκB staining (nuclear labeling index, NLI) is shown. Infected HbAA erythrocytes (iHbAA) were pre-incubated with purified ICAM-1 and/or CD36 protein for 1 hr prior to the interaction with HDMEC. Erythrocytes infected with a knobless FCR3 line (K-) were investigated in parallel. Untreated iHbAA and TNF-α served as a positive control, whereas uninfected HbAA erythrocytes (HbAA) and medium were used as negative controls. A box plot analysis is overlaid over the individual data points, with the median, 25% and 75% quartile ranges shown for three independent biological replicates. Statistical significance was assessed, using Holm-Sidak one-way ANOVA. a.u., arbitrary unit. **b** The levels of IL-6 secretion and (**c**) ICAM-1 endothelial surface expression were quantified by ELISA and flow cytometry, respectively, after co-culturing confluent HDMEC with infected erythrocytes for 20 hrs. Medium and uninfected red blood cells served as negative controls, whereas TNF-α served as a positive control. Trans-well assays (trans-well) were used to prevent direct contact between endothelial cells and infected erythrocytes, but allowed the soluble factors to be exchanged. In (**b**) A box plot analysis is overlaid over the individual data points, with the median, 25% and 75% quartile ranges shown for three independent biological replicates. A representative example of three independent biological replicates is shown in (**c**).
