# Supplementary material for: The sickle cell trait affects contact dynamics and endothelial cell activation in Plasmodium falciparum-infected erythrocytes
Source: Commun Biol. 2018 Nov 30;1:211. doi: 10.1038/s42003-018-0223-3 (PMC6269544; doi:10.1038/s42003-018-0223-3)
Supplement: Supplementary file 3 — Description of Additional Supplementary Files [file 42003_2018_223_MOESM3_ESM.docx]

**Description of Additional Supplementary Files**

**File Name**: Supplementary Movie 1

**Description**: Representative fluorescence microscopy movie showing the motion behavior of an infected HbAA erythrocyte at the trophozoite stage over a confluent monolayer of human dermal microvascular endothelial cells. The typical flipping behavior is observed. The trajectories derived from this cell are shown in Fig. 1c. Wall shear stress 0.03 Pa; scale bar 20 µm.

**File Name**: Supplementary Movie 2

**Description**: Representative fluorescence microscopy movie showing the motion behavior of an infected HbAA erythrocyte at the schizont stage over a confluent monolayer of human dermal microvascular endothelial cells. The typical smooth rolling behavior is observed. The trajectories derived from this cell are shown in Fig. 1d. Wall shear stress 0.03 Pa; scale bar 20 µm.

**File Name**: Supplementary Movie 3

**Description**: Representative fluorescence microscopy movie showing the motion behavior of an infected HbAS erythrocyte at the trophozoite stage over a confluent monolayer of human dermal microvascular endothelial cells. The typical rough flipping behavior is observed. The trajectories derived from this cell are shown in Fig. 1e. Wall shear stress 0.03 Pa; scale bar 20 µm.

**File Name**: Supplementary Movie 4

**Description**: Representative fluorescence microscopy movie showing the motion behavior of an infected HbAS erythrocyte at the schizont stage over a confluent monolayer of human dermal microvascular endothelial cells. The typical rough rolling behavior is observed. The trajectories derived from this cell are shown in Fig. 1f. Wall shear stress 0.03 Pa; scale bar 20 µm.

**File Name**: Supplementary Movie 5

**Description**: Representative phase contrast movie of an infected HbAA erythrocyte at trophozoite stage, showing the typical flipping behavior. The snap shots depicted in Fig. 3b are derived from this movie. Wall shear stress 0.03 Pa; scale bar 20 µm.

**File Name**: Supplementary Movie 6

**Description**: Representative phase contrast video of an infected HbAA erythrocyte at the schizont stage, showing the typical smooth rolling behavior. The snap shots depicted in Fig. 3b are derived from this movie. Wall shear stress 0.03 Pa; scale bar 20 µm.

**File Name**: Supplementary Movie 7

**Description**: Simulated dynamic adhesion behavior of a *P. falciparum*-infected erythrocyte at the trophozoite stage. The snap shots depicted in Fig. 3b are derived from this movie.

**File Name**: Supplementary Movie 8

**Description**: Simulated dynamic adhesion behavior of a *P. falciparum*-infected erythrocyte at the schizont stage. The snap shots depicted in Fig. 3b are derived from this movie.

**File Name**: Supplementary Movie 9

**Description**: Representative fluorescence microscopy movie showing the motion behavior of an infected HbAA erythrocyte at the trophozoite stage over a confluent monolayer of human dermal microvascular endothelial cells. The typical flipping behavior is observed. The trajectories derived from this cell are shown in Supplementary Fig. 5a. Wall shear stress 0.03 Pa; scale bar 20 µm.

**File Name**: Supplementary Movie 10

**Description**: Representative fluorescence microscopy movie showing the motion behavior of an infected HbAA erythrocyte at the schizont stage over a confluent monolayer of human dermal microvascular endothelial cells. The typical smooth rolling behavior is observed. The trajectories derived from this cell are shown in Supplementary Fig. 5b. Wall shear stress 0.03 Pa; scale bar 20 µm.

**File Name**: Supplementary Movie 11

**Description**: Representative fluorescence microscopy movie showing the motion behavior of an infected HbAS erythrocyte at the trophozoite stage over a confluent monolayer of human dermal microvascular endothelial cells. The typical rough flipping behavior is observed. The trajectories derived from this cell are shown in Supplementary Fig. 5c. Wall shear stress 0.03 Pa; scale bar 20 µm.

**File Name**: Supplementary Movie 12

**Description**: Representative fluorescence microscopy video showing the motion behavior of an infected HbAS erythrocyte at the schizont stage over a confluent monolayer of human dermal microvascular endothelial cells. The typical rough rolling behavior is observed. The trajectories derived from this cell are shown in Supplementary Fig. 5d. Wall shear stress 0.03 Pa; scale bar 20 µm.

**File Name**: Supplementary Data 1

**Description**: The excel file contains the source data underlying the graphs presented in the main article and supplementary information.
